# Administration of *Lactobacillus reuteri* Combined with *Clostridium butyricum* Attenuates Cisplatin-Induced Renal Damage by Gut Microbiota Reconstitution, Increasing Butyric Acid Production, and Suppressing Renal Inflammation

**DOI:** 10.3390/nu13082792

**Published:** 2021-08-15

**Authors:** Yu-Ping Hsiao, Hsiao-Ling Chen, Jen-Ning Tsai, Meei-Yn Lin, Jiunn-Wang Liao, Meng-Syuan Wei, Jiunn-Liang Ko, Chu-Chyn Ou

**Affiliations:** 1 Institute of Medicine, Chung Shan Medical University, Taichung 40201, Taiwan; missyuping@gmail.com; 2 Department of Dermatology, Chung Shan Medical University Hospital, Taichung 40201, Taiwan; 3 Department of Food, Nutrition and Health Biotechnology, Asia University, Taichung 41354, Taiwan; hlchen908@asia.edu.tw; 4 Department of Medical Laboratory and Biotechnology, Chung Shan Medical University, Taichung 40201, Taiwan; jeningts@csmu.edu.tw; 5 Clinical Laboratory, Chung Shan Medical University Hospital, Taichung 40201, Taiwan; 6 Department of Food Science and Biotechnology, National Chung Hsing University, Taichung 40227, Taiwan; mylin@dragon.nchu.edu.tw (M.-Y.L.); priscillawie@gmail.com (M.-S.W.); 7 Graduate Institute of Veterinary Pathobiology, National Chung Hsing University, Taichung 40227, Taiwan; jwliao@dragon.nchu.edu.tw; 8 Department of Medical Oncology and Chest Medicine, Chung Shan Medical University Hospital, Taichung 40201, Taiwan; 9 Department of Nutrition, Chung Shan Medical University Hospital, Taichung 40201, Taiwan; 10 Department of Nutrition, Chung Shan Medical University, Taichung 40201, Taiwan

**Keywords:** cisplatin, nephrotoxicity, *Clostridium butyricum*, *Lactobacillus reuteri*, *Escherichia-Shigella*

## Abstract

Cisplatin-induced nephrotoxicity is associated with gut microbiota disturbance. The present study aimed to investigate whether supplementation of *Lactobacillus reuteri* and *Clostridium butyricum* (LCs) had a protective effect on cisplatin-induced nephrotoxicity through reconstruction of gut microbiota. Wistar rats were given different treatments: control, cisplatin (Cis), cisplatin + *C. butyricum* and *L. reuteri* (Cis+LCs), and *C. butyricum* and *L. reuteri* (LCs). We observed that cisplatin-treated rats supplemented with LCs exhibited significantly decreased renal inflammation (KIM-1, F4/80, and MPO), oxidative stress, fibrosis (collagen IV, fibronectin, and a-SMA), apoptosis, concentration of blood endotoxin and indoxyl sulfate, and increased fecal butyric acid production compared with those without supplementation. In addition, LCs improved the cisplatin-induced microbiome dysbiosis by maintaining a healthy gut microbiota structure and diversity; depleting *Escherichia-Shigella* and the *Enterobacteriaceae* family; and enriching probiotic *Bifidobacterium*, *Ruminococcaceae*, *Ruminiclostridium_9*, and *Oscillibacter*. Moreover, the LCs intervention alleviated the cisplatin-induced intestinal epithelial barrier impairment. This study indicated LCs probiotic serves as a mediator of the gut–kidney axis in cisplatin-induced nephrotoxicity to restore the intestinal microbiota composition, thereby suppressing uremic toxin production and enhancing butyrate production. Furthermore, the renoprotective effect of LCs is partially mediated by increasing the anti-inflammatory effects and maintaining the integrity of the intestinal barrier.

## 1. Introduction

Cisplatin is widely used as an effective chemotherapeutic agent for many carcinomas; however, it can lead to adverse side effects on the kidney and gastrointestinal tract [1,2], thereby limiting its use and efficacy in cancer therapy. Chemotherapy is usually accompanied with distinctly altered gut bacterial composition known as dysbiosis [3,4,5]. Dysbiosis is defined by less of the good microbes such as lactobacilli/bifido species and more of the pathogens. Many studies have suggested that cisplatin-induced nephrotoxicity is closely related to an alteration of gut microbiota composition, destruction of the intestinal barrier, and accumulation of uremic substances [1,2].

Considerable data have shown that modification of the intestinal flora through probiotics can alleviate kidney disease progression [2,6,7]. The mechanisms of action of probiotics with health-beneficial effects remain unknown, but probiotics can modulate the gut microbiota and suppress the growth of pathogenic microorganisms. *Clostridium butyricum* strain is a strict anaerobic, sporulating bacterium that is usually used to clinically treat patients with mild diarrhea, abdominal pain, and constipation, which affects adjustment of bowel movements and soft stools [8]. *Clostridium butyricum* MIYAIRI 588 (CBM588)-formed endospores could prevent gastric acid in the intestinal tract, and it subsequently enters the colon where it germinates, proliferates, and produces short-chain fatty acids (SCFAs), particularly butyric acid [9]. Several studies have shown that CBM588 exerts intestinal anti-inflammatory effects on experimental models of intestinal inflammation [10,11], which is associated with IL-10 production [10]. CBM588 is safe, and it has anticancer effects on bladder cancer in vitro and in vivo [12]. Moreover, *C. butyricum* suppresses the development of intestinal tumors partly by modulating Wnt/β-catenin signaling and gut microbiota and increasing SCFA production [13].

Recently, some mechanisms of the beneficial effects of *L. reuteri* in promoting health and preventing infections and diverse diseases have been proposed: (1) producing antimicrobial molecules, such as organic acids, ethanol, and reuterin to inhibit the colonization of pathogenic microbes and reconstruction of the commensal microbiota composition in the host; (2) enhancing the immune system by promoting regulatory T cell development and function; and (3) enhancing mucosal barrier function [14].

*L. reuteri* inhibits the development of mammary tumors through stimulating host immune cells in westernized diet-induced or genetic predilection-induced breast cancer [15]. The metabolic impairments caused by a high-fat diet are alleviated by supplementation with *L. reuteri* FN041, which is associated with improvement of the intestinal epithelial barrier, gut microbiota restoration, and SCFA production [16]. However, *L. reuteri* has been reported to have a protective effect on colitis caused by dextran sodium sulfate (DSS), but it does not maintain the integrity of the mucus layer or prevent dysbiosis [17].

In clinical practice, *L. reuteri* [18] and *C. butyricum* [8] have been used for several years, but the potential protective effect and mechanisms involving both strains on cisplatin-induced side effects (nephrotoxicity and intestinal mucositis) remain unclear. Based on our previous analyses [5] and other study findings [1], the pathophysiology of intestinal mucositis caused by cisplatin may be associated with disrupted gut microbiome homeostasis. Modulation of intestinal microbiota by administering probiotics could be considered as a beneficial intervention to remove uremic toxins, promote butyrate production, and improve renal function [2,19]. We hypothesize that LCs supplementation may prevent cisplatin-induced kidney damage progression by restoring the environment of gut microbiota and activating anti-inflammatory activity. Here, we identified the role of probiotics as a mediator of the gut–kidney axis in cisplatin-induced nephrotoxicity. The renoprotective effect of LCs probiotic is mediated by the modulation of the intestinal microbiota composition, thereby suppressing uremic toxin production and enhancing butyrate production. Furthermore, LCs modulate gut microbiota composition to inhibit pathogenic species (such as endotoxin-secreting bacteria) and reduce gut-driven endotoxin production, which ultimately mitigate cisplatin-induced intestinal toxicity.

## 2. Material and Methods

### 2.1. Animals

The experimental protocol was approved by the Affidavit of Approval of Animal Use Protocol, Chung Shan Medical University Experimental Animal Center, Taichung, Taiwan (Approval No: 2066).

### 2.2. Probiotic Treatment

*L. reuteri* and *C. butyricum* probiotic strains, which were studied in this work, were selected on the basis of the abundance of gut microbiota in a cisplatin-induced nephrotoxicity model during D-methionine coadministration [5]. In addition, both strains provided beneficial nutrients to the host [20] and they have shown anti-oxidant and anti-inflammatory effects in the intestine [11,21,22,23].

The probiotic mixtures used in this study were *L. reuteri* and *C. butyricum* (Miyarisan Pharmaceutical). *L. reuteri* (BCRC 80379) was obtained from the Food Industry Research and Development Institute (Hsinchu, Taiwan), whereas CBM588 was purchased from Asia Bio-Med Management Limited (Taichung, Taiwan). The probiotic mixtures contained viable CBM588 at 5 × 10^8^ CFU/g and viable *L. reuteri* at 1–2 × 10^9^ CFU/mL.

### 2.3. Study Design

Male Wistar rats were purchased from BioLASCO Taiwan Co., Ltd. and randomly divided into four groups. The rats were given different treatments: control, cisplatin (Cis), cisplatin + *C. butyricum* and *L. reuteri* (Cis+LCs), and *C. butyricum* and *L. reuteri* (LCs). Wistar rats received LCs by oral gavage for 10 days prior to cisplatin induction. Cisplatin (5 mg/kg; i.p.) was induced once a week for four weeks. Combined LCs treatment was received for 24 days, then the rats were sacrificed at the 25th day. 

On days 23 and 24, the fecal samples in all rats were collected into sterile containers, weighed, and dried in an oven at 50 °C for 72 h to measure moisture content. Part of the fecal pellets was collected to determine fecal pH using a pH meter and to measure SCFAs, and cecum contents were used for NGS analysis. On day 25, the animals were sacrificed. Blood samples were centrifuged, and serum were stored at −80 °C before biochemical analysis. Then, the intestine, stomach, spleen, kidney, and liver were quickly weighed and then immediately divided into two portions: one was kept in 10% formalin for histopathological investigation and the other was stored at −80 °C until analysis. The colons were removed, and colon length was measured using a ruler. 

### 2.4. Measurement of Serum Renal Function and Biochemical Parameters

The reagents of biochemical analyses used in this study were obtained from Sentinel using the automated chemical analyzer (TBA-120FR, Toshiba Medical Systems, Inc., Tokyo, Japan). Blood Cystatin C and IgA were measured using turbidimetric immunology (Cystatin C, Latex, Minneapolis, MN, USA) according to the manufacturer’s instructions. In addition, urea nitrogen was measured using urease with GLDH (Coupled Enzymes); AST and ALT were measured using kinetic JSCC; and uric acid was measured using uricase peroxidase according to the manufacturer’s instructions. Creatinine was determined in the serum by colorimetry using a commercial kit (Randox Laboratories Ltd., Crumlin, UK), according to the manufacturer’s protocols.

### 2.5. Preparation of Tissue Homogenates

Approximately 0.1 g of the intestine and kidney was homogenized separately in cold PBS buffer using a homogenizer. The crude tissue homogenate was then centrifuged, and the supernatant was kept at −80 °C.

### 2.6. Oxidative Stress Biomarker Analysis

The level of malondialdehyde (MDA) in kidney homogenates was measured after reaction with thiobarbituric acid. Glutathione peroxidase (GPx) was measured by monitoring the oxidation of GSH coupled to nicotinamide adenine dinucleotide phosphate by glutathione reductase (GSR) as described previously [24]. Catalase activity assay was based on the rate of decrease in hydrogen peroxide at 240 nm. Another part of the kidney supernatants was used to measure IL-10 production by ELISA kits (R & D Systems, Minneapolis, MN, USA). 

### 2.7. Serum Endotoxin, Myeloperoxidase, Kidney Homogenate Myeloperoxidase, and Hydroxyproline Measurements

Serum endotoxin concentrations were measured using Endotoxin Quant kit (ThermoFisherA39552, Rockford, IL, USA) according to the manufacturer’s instructions. The serum and kidney homogenates myeloperoxidase (MPO) activity, as an indirect index of neutrophil infiltration, was determined using the previously described procedure with some modification [25]. In addition, another portion of kidney homogenates was measured using the hydroxyproline colorimetric assay kit (K555, BioVision, Milpitas, CA, USA) following the manufacturer’s protocols.

### 2.8. Indoxyl Sulfate (IS) Assay

Plasma deproteinization was performed by adding methanol containing IS (as internal standards) to plasma. Then, samples were mixed vigorously and centrifuged. The collected supernatant was transferred to an autosampler (San Jose, CA, USA) and injected into a LC/MS system (Thermo Scientific, Milford, CT, USA).

### 2.9. Protein Extraction and Western Blot Analysis

The kidney tissue was homogenized in homogenization buffer (containing proteinase inhibitor) by ultrasonic treatment. The protein concentrations were measured through Bradford protein assay (Bio-Rad protein assay reagent (500-0006, Bio-RAD, Hercules, CA, USA)) with bovine serum albumin (sc-2323, Bio-RAD, Hercules, CA, USA) standards. For Western blot analysis, 50 μg of protein was separated by SDS-PAGE, transferred to a nitrocellulose membrane, blocked with 5% nonfat milk, and then stained with α-SMA, β-catenin, fibronectin, and KIM-1 primary antibodies (diluted 1:1000). The membranes were further incubated with a horseradish peroxidase conjugated secondary antibody. The blots were washed with PBS-Tween and then visualized by enhanced chemiluminescence (NML104001EA, Waltham, MA, USA).

### 2.10. Real-Time PCR

Tight junction protein zonula occludens-1 (ZO-1) were analyzed using a real-time PCR system. Total RNA was extracted from the ground powder of intestinal tissues using 1 mL of RareRNA reagent following the manufacturer’s protocol. Quantification of RNA was performed using the Amersham-Ultrosrec 2100 Pro spectrophotometer (Biochrom, Cambridge, UK). Three micrograms of purified RNA were reverse transcribed to cDNA using high-capacity cDNA reverse transcription kits (Applied Biosystem, Part Number: 4368813) and oligo (dT) primers and subjected to PCR according to the manufacturer’s instructions. All the PCR reactions were performed in an MJ Peltier Thermal cycle PTC-200 (MJ Research, Watertown, MA, USA) in a total volume of 25 µL of reaction mixture. Quantitative real-time polymerase chain reaction was carried out using the SYBR green system and the StepOneTM Real-Time PCR System (Applied Biosystems). GAPDH, a common housekeeping gene, was selected as an internal control. Relative mRNA expression was calculated according to the ΔΔCT method. The results were expressed as relative gene expression normalized to the expression levels of the endogenous GAPDH.

The PCR-primer sequences were as follows: ZO-1 (forward) 5′-CCATCTTTGGACCGATTGCTG-3′ and (reverse) 5′-TAATGCCCGAGCTCCGATG-3′.

### 2.11. Histopathological Examination

Kidney and intestinal (duodenum, jejunum, and ileum) segments were fixed with 4% paraformaldehyde for 24 h. Slides of kidney and intestinal were subsequently stained with hematoxylin/eosin (H&E) for microscopic examination. The severity of tissue lesions was graded according to the methods described by Shackelford et al. [26]. In addition, 5 μm kidney or ileum sections were cut for microtomy, followed by staining with Masson’s trichrome (MT) stain to identify the fibrous connective tissues and periodic acid Schiff’s (PAS) stain for the detection of mucopolysaccharides to observe the atrophy of the basal membrane and dilatation. The ileum sections were also stained with PAS to observe changes in goblet cell mucin stores.

### 2.12. Immunohistochemistry (IHC) Analysis

For IHC, the specimens were processed under stander protocol and stained with primary antibodies: F4/80 Polyclonal Antibody (Thermo Fisher), Cleaved Caspase-3 antibody (9661, Cell signal, Danvers, MA, USA), and collagen IV Antibody (ab6586, Abcam, Cambridge, MA, USA) overnight at 4 °C. Sections were then incubated sequentially with secondary antibody (goat anti-rabbit or rabbit anti-rat). The positive results were indicated by brown coloration of antigen-containing cells. Finally, Image Pro 6.2 software was used to quantify the positive staining areas of F4/80, Caspase-3, and collagen IV in 10 randomly selected sections of the kidney cortex using ×40 magnification.

### 2.13. Measurement of Brush Border Membrane (BBM) Enzyme

The activities of BBM biomarkers, such as enzymes, sucrase, maltase, lipase, leucine, aminopeptidase (LAP), in the mucosal homogenates were determined. LAP was assayed using l-leucine *p*-nitroanilide as substrate. Sucrase was assayed using the reduced sugars formed upon the hydrolysis of sucrose. The activities of sucrase, lipase, and LAP were measured using the Randox assay kit (Randox Laboratories, Crumlin, UK).

### 2.14. Short-Chain Fatty Acid Measurement

Fecal acetate, propionate, and butyrate were determined with gas chromatography. Fecal samples were extracted using methyl ether with 4-methyl-*n*-valeric acid (Sigma) as an internal standard according to the method described previously [27]. The extracts were dissolved in 1 M of phosphoric acid before being injected into the gas chromatograph (GC-2014; Shimadzu Corp., Kyoto, Japan) fitted with a glass capillary column (0.25 mm × 30 m, Stabilwax-DA, Restek Corp., Bellefonte, PA, USA) and a flame ionization detector. The flow rate of the carrier (N2) was 1.5 mL min^−1^. 

### 2.15. 16s rDNA Sequencing and Bioinformatics Analysis

Total genome DNA from rat cecum content was extracted using commercial DNA Stool Mini extraction kit (Qiagen GmbH) according to the manufacturer’s protocols. The V3 and V4 hypervariable regions of prokaryotic 16S rDNA were selected for generating amplicons following taxonomy analysis. The sequence length was larger than 200 bp sequence. After quality filtering, chimeric sequences, the resulting sequence for operational taxonomic unit (OTU) clustering, were purified using VSEARCH clustering (1.9.6) sequence (sequence similarity is set to 97%), and then Silva 132 was used as the 16 s rRNA reference database. Based on the OTU analysis results, random sampling of sample sequences was flat, and Chaol (community richness), Shannon (community diversity), and observed species (estimated OTU amounts) were calculated. Alpha diversity was also estimated using the phylogenetic diversity metric. Beta diversity analysis was used to compare gut microbiota compositions among the groups and performed using UPGMA clustering based on weighted and unweighted UniFrac distances. Principal coordinate analysis (PCoA) was based on the distance matrix. Weighted UniFrac and Unweighted UniFrac were calculated to assist PCoA. PCoA was conducted and displayed using Qiime software (Version1.7.0). The Linear Discriminant Analysis Effect Size (LEfSe) analysis was used for the quantitative analysis of biomarkers within different groups. MetagenomeSeq was used to determine differentially abundant taxa among the four treatment groups with adjusted *p*-value cut off at 0.05.

## 3. Statistical Analyses

Data were expressed as mean ± SEM. Statistical differences were determined using one-way test and post hoc test. *p* values less than 0.05 were considered significant among groups. Statistical analysis for gut microbiota, Mann-Whitney test, Kruskal-Wallis test, and ANOVA were used to evaluate the differences in the bacterial populations among groups.

## 4. Results

### 4.1. Pre-Administration of LCs Ameliorates Cisplatin-Induced Gastrointestinal Toxicity

Anorexia and loss of body weight were major clinical adverse effects during therapy with cisplatin [2,28]. We recorded the changes of food intake and body weight daily to investigate the benefit of LCs on appetite and body weight gain. The levels of food intake, body weight, feeding efficiency ratio (FER), and gastric emptying index in the cisplatin group were significantly lower than that in the control group, whereas the value of gastric contents was significantly higher than that in the control group. Compared with the cisplatin group, these changes of digestive parameters were significantly reversed by LCs administration (Supplementary Figure S1A–E). These results indicated that LCs attenuated cisplatin-induced gastrointestinal toxicity in rats. LCs administration alone did not alter body weight, food intake, or gastrointestinal function during the experimental period.

### 4.2. LCs Ameliorated Cisplatin-Induced Nephrotoxicity

The ratio of kidney/body weight in the cisplatin group was significantly higher than that in the control group (*p* < 0.05), but it significantly decreased after supplementation with LCs. The ratio of liver/body weight in the Cis+LCs and LCs groups was significantly lower than that in the cisplatin group, whereas the ratio of stomach/body weight in the Cis+LCs group was significantly higher than that in the cisplatin group (Supplementary Table S1).

Apart from creatinine and BUN (traditional markers of kidney function), the elevated cystatin C concentration was considered as an indicator of renal functional impairment [29]. The levels of creatinine, BUN, Cystatin C, IgA, and IS in the cisplatin group were significantly higher than that in control group, whereas the value of uric acid was significantly lower than that in the control group. Compared with the cisplatin group, these changes in blood biochemistry indexes were significantly reversed by LCs administration (Table 1). LCs alone treatment did not alter kidney function markers. The levels of AST were not significantly different among the four groups. In particular, ALT concentration was markedly lower in the Cis +LCs group and LCs group than that in the cisplatin group.

### 4.3. Effects of LCs on Histopathological Changes in the Kidneys of Cisplatin-Induced Nephrotoxicity Rats

**Figure 1:**
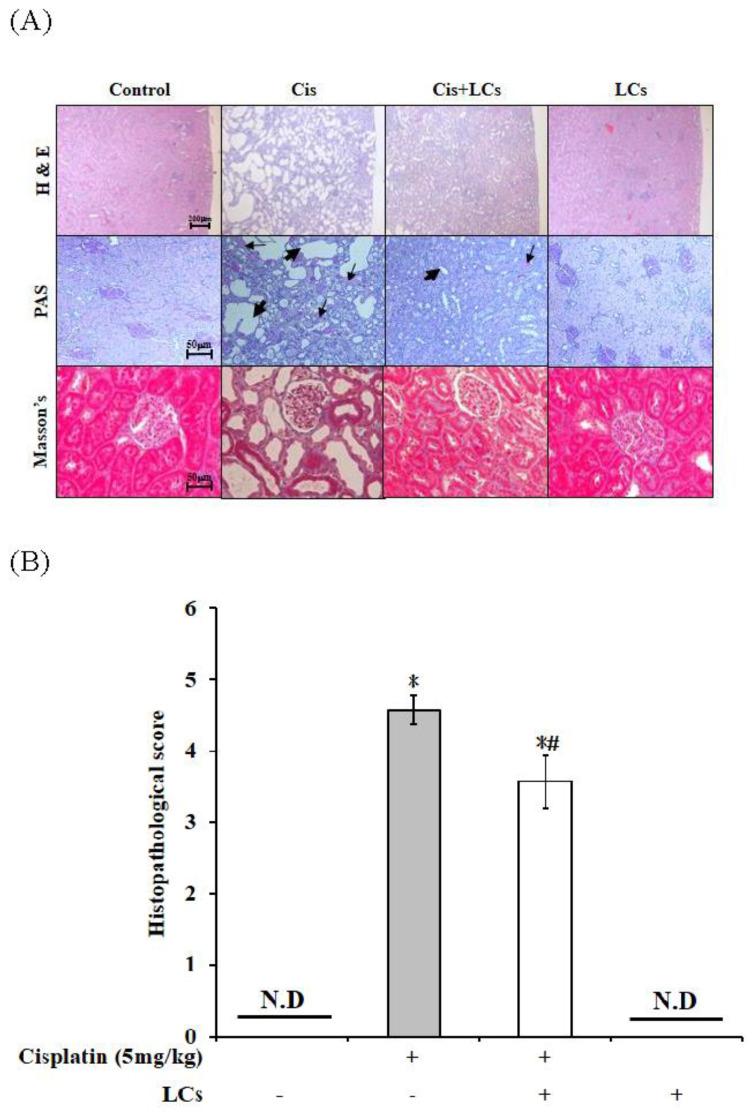
Histology of the kidney sections in a cisplatin-induced nephrotoxicity model. (**A**) H&E staining, periodic acid Schiff staining (PAS), and Masson’s trichrome staining (MT). (**B**) Quantitative analysis of histopathological kidney lesions. Results are shown as mean ± SEM (*n* = 7 per group). * *p* < 0.05 compared with the control group; # *p* < 0.05 compared with the cisplatin group.

The kidney function was restored by LCs. Next, we investigated the effect of LCs on cisplatin-induced histological damages using H&E staining. The morphological analyses of the kidney sections showed that cisplatin-treated rats exhibited a severe histological injury such as tubule dilatation, cast formation, inflammatory cell infiltration, renal interstitial fibrosis, and renal tubular swollenness (Figure 1A). The kidney tissue morphology damages caused by cisplatin were attenuated by LCs. The highest kidney injury score was found in cisplatin-treated rats and was evidently improved after LCs intervention (Figure 1B) (*p* < 0.05), suggesting that LCs could prevent cisplatin-induced nephrotoxicity.

**Figure 2:**
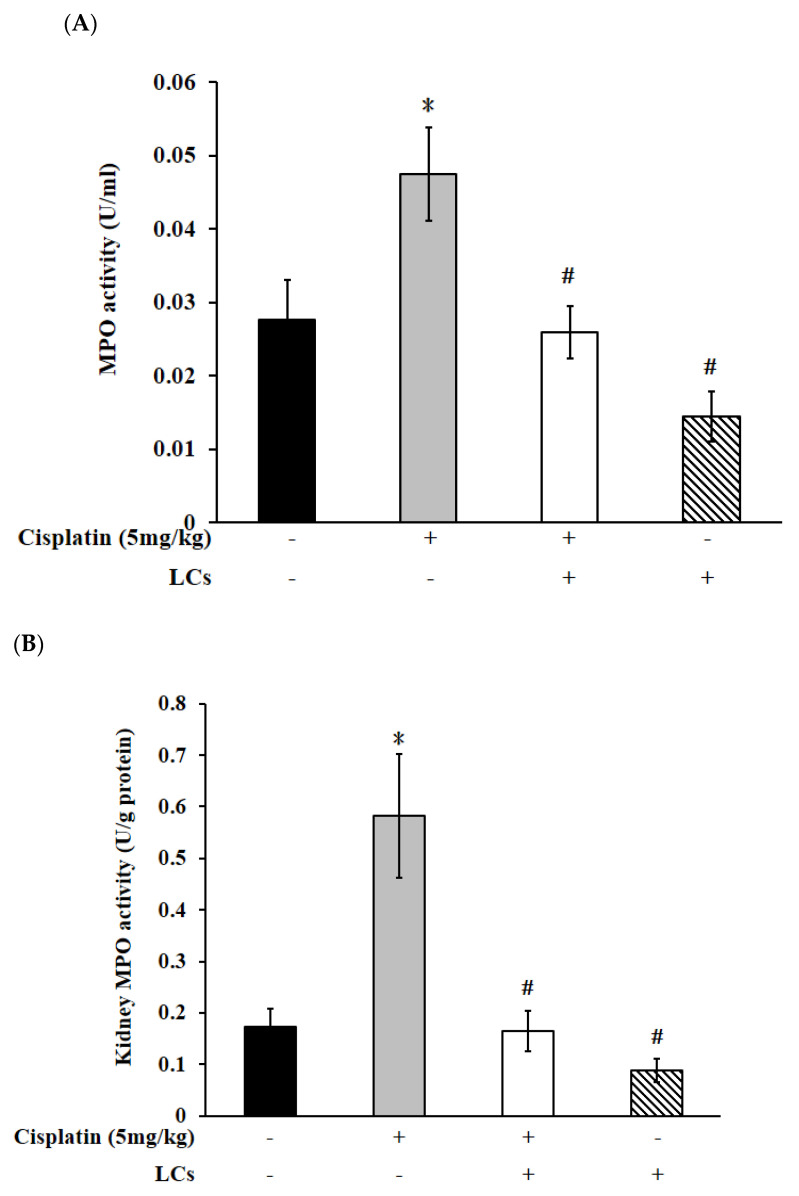

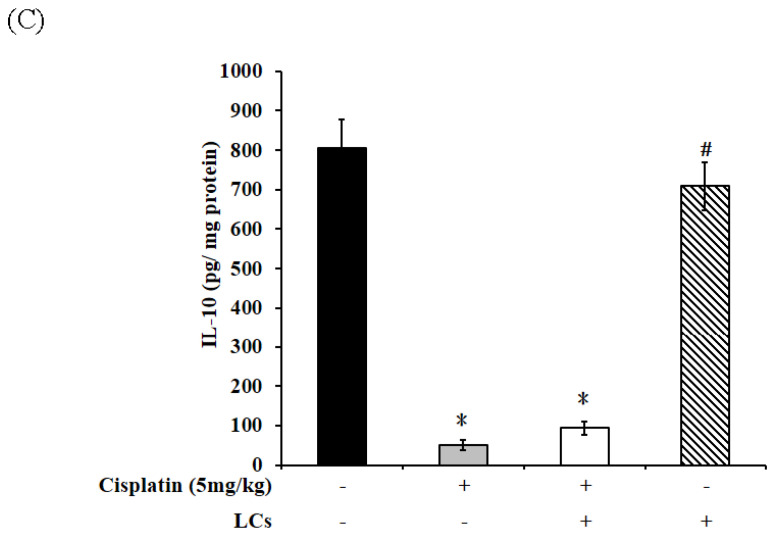
Effects of cisplatin and LCs supplementation on inflammation responses in kidney tissues. (**A**) Serum MPO. (**B**) Kidney MPO. (**C**) IL-10. Results are shown as mean ± SEM (*n* = 7 per group). * *p* < 0.05 compared with the control group; # *p* < 0.05 compared with the cisplatin group.

In general, during MT staining, collagen accumulated around renal tubules appeared light blue, which was considered as a hallmark of tissue fibrosis [30]. In our study, Figure 2 shows that the rats multiple-injected with 5 mg/kg of cisplatin once a week for four times showed significant tubulointerstitial fibrosis of the kidney sections, which was consistent with published results by Li et al. [30]. Loss of basement membranes (abundant cytoplasmic mucopolysaccharides) after cisplatin treatment was observed by PAS staining in renal tubular epithelial cells. The above-mentioned histopathological changes were reversed by LCs administration in cisplatin-treated rats. LCs pre-administration was effective in preventing the progression of renal fibrosis.

### 4.4. Effects of LCs on Renal Oxidative Stress Parameters, MPO, and IL-10 Level in the Cisplatin-Induced Nephrotoxicity Model

Compared with the control group, the MDA contents increased, and catalase and GPx activity decreased because of cisplatin treatment, whereas only MDA levels were significantly decreased by LCs supplementation. Catalase and GPx activity were slightly restored, but they were not statistically significant after LCs intervention (Supplementary Figure S2A–C). MPO levels were considered as an indicator of neutrophil infiltration into tissues [31]. Cisplatin resulted in increased MPO levels in kidney tissue and serum (*p* < 0.05; Figure 2A,B), which were remarkably declined through supplementation with LCs. Rats treated with cisplatin exhibited a decrease in IL-10 content in kidney tissue as compared with the control group, which was slightly increased when they were fed with LCs, but the increase was not statistically significant (Figure 2C).

### 4.5. LCs Attenuates Kidney Inflammation, Apoptosis, and Fibrosis in Cisplatin-Induced Nephrotoxicity Models

**Figure 3:**
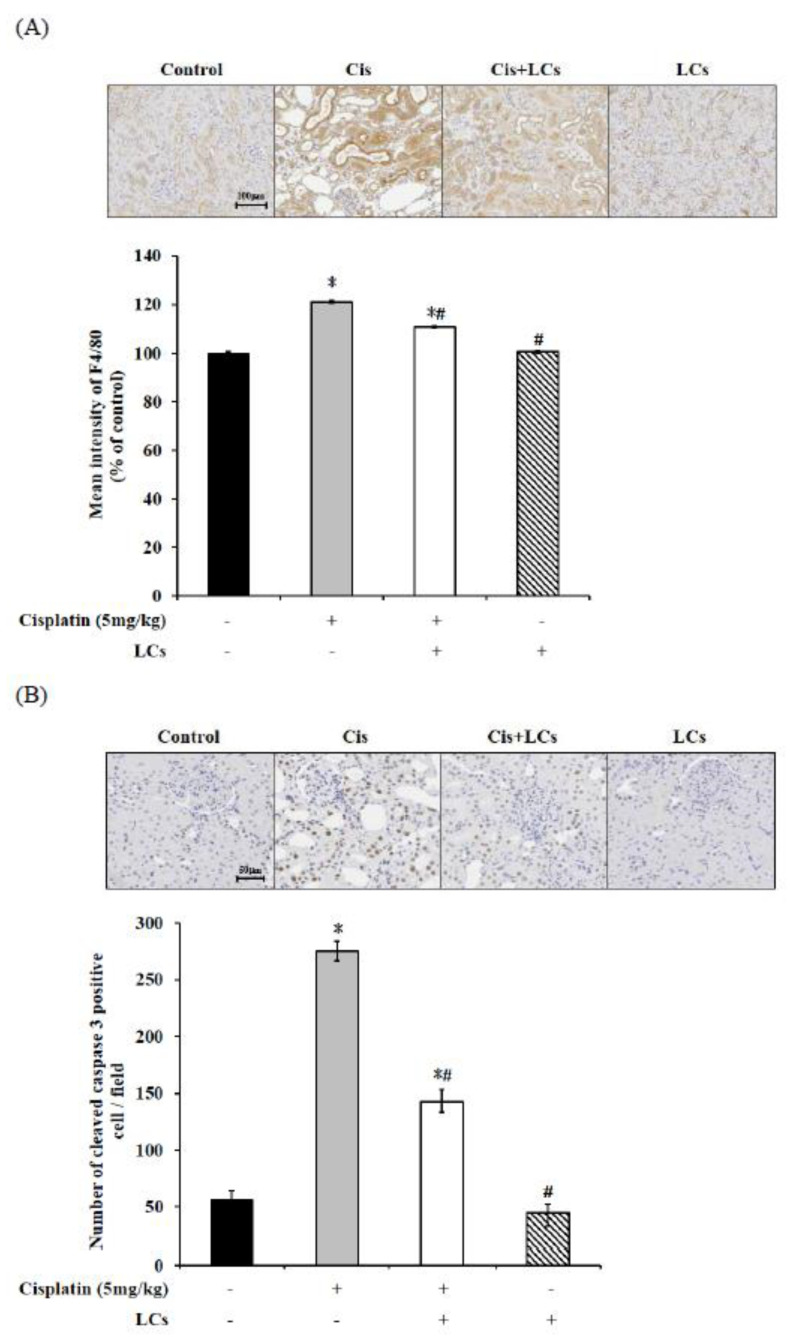

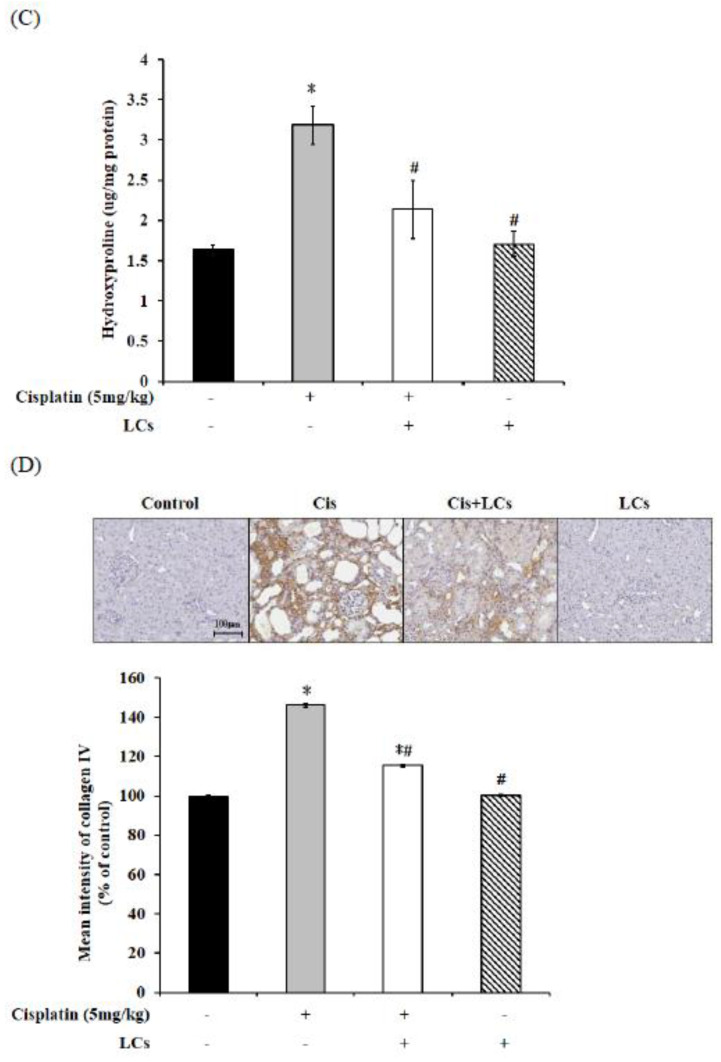

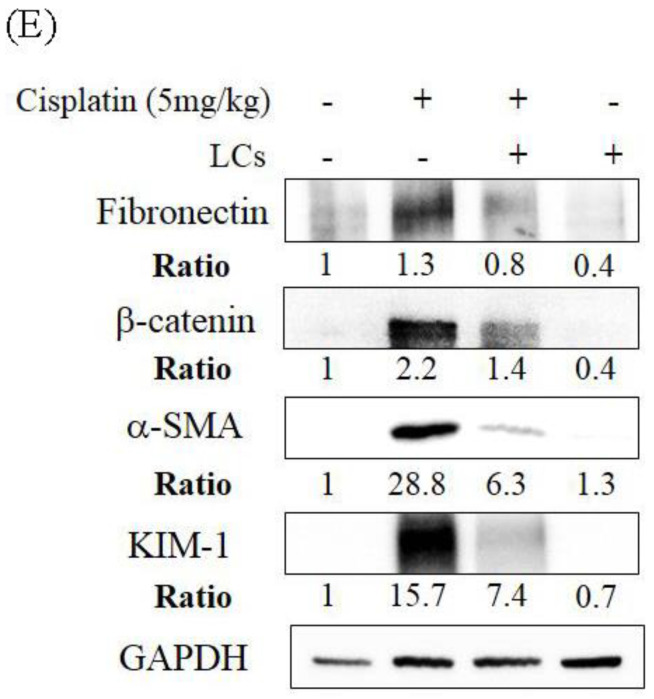
LCs supplementation alleviates renal fibrosis, necrosis, and macrophage infiltration in cisplatin-induced nephrotoxicity rats. (**A**) Immunohistochemistry shows F4/80^+^ macrophage infiltration and quantitative analysis. (**B**) Immunohistochemistry shows cleaved caspase-3-positive cells and quantitative analysis of tubular necrosis. (**C**) Hydroxyproline levels. (**D**) Immunohistochemistry shows collagen IV intensity and quantitative analysis of positive cells. (**E**) Western blot analysis shows renal fibronectin, β-catenin, α-SMA, and KIM-1 protein expression. Results are shown as mean ± SEM (*n* = 7 per group). * *p* < 0.05 compared with the control group; # *p* < 0.05 compared with the cisplatin group.

As shown in Figure 3A, cisplatin alone treatment markedly increased the density of F4/80-expressing cells, suggesting increased infiltration of macrophages.

However, LCs co-administration after cisplatin treatment reduced F4/80 protein expression, which indicated that LCs could prevent macrophage infiltration. 

Caspase-3-positive apoptotic cells (indicated by arrows) were increased in the kidney after cisplatin treatment and were greatly attenuated by LCs administration (Figure 3B). KIM-1 is a transmembrane glycoprotein, which is normally undetectable in the normal kidney but thoroughly expressed in proximal tubule cells after toxicant injury. Figure 3E shows that treatment with cisplatin also significantly increased kidney KIM-1 expression in comparison with control animals. Administration of LCs reduced the protein level of KIM-1 in the damaged kidney.

We further evaluated some molecular and histological markers of fibrosis to understand the antifibrotic effects of LCs in tubule injury. Hydroxyproline may be a potential marker of nephrotoxicity related to fibrosis following dosing with cisplatin [32]. We also found that the high values of kidney hydroxyproline in rats treated with cisplatin were consistent with the occurrence of fibrosis and renal tubule necrosis noted in the histopathological results (Figure 3C). Meanwhile, quantification of collagen IV-area fraction showed higher expression values of collagen IV-positive cells in cisplatin treatment groups compared with the control group (Figure 3D). Further analysis using Western blot demonstrated significantly higher expression of fibronectin, β-catenin, and α-SMA protein in the kidney of the cisplatin treatment group compared with the control group. Meanwhile, an improved histological fibrosis was exhibited in the LCs supplementation group, which had a low level of inflammation cell infiltration, collagen IV-positive cells, and expression of fibronectin, β-catenin, and α-SMA protein in kidney tissues as compared with the cisplatin group (Figure 3E). Therefore, LCs administration could reduce cisplatin-induced fibrosis.

### 4.6. LCs Promote the Output of Stool in Rats Treated with Cisplatin

We have previously shown that the amounts of stool were significantly decreased, and the appearance of cecum contents was watery in cisplatin-treated rats [5]. Supplementary Table S2 shows that the total amounts of stool in the cisplatin group were significantly reduced compared with the other three groups, which were significantly increased when they were fed with LCs (*p* < 0.05). In addition, no significant change in the amounts of stool was observed between the LCs alone group and control group. Especially, highest levels of the stool moisture content were observed in the LCs alone group among four groups. No significant differences in the stool moisture content and pH value were observed among the experimental groups (*p* < 0.05). We also observed that the cecum contents of rats treated with cisplatin were watery, but soft stools were observed in rats treated with cisplatin after LCs intervention, and a higher proportion of formed and hard stools was found in the control and LCs groups.

### 4.7. LCs Intervention Alleviates Intestinal Damage and Improves Shortened Colon Length in Cisplatin-Treated Rats

**Figure 4:**
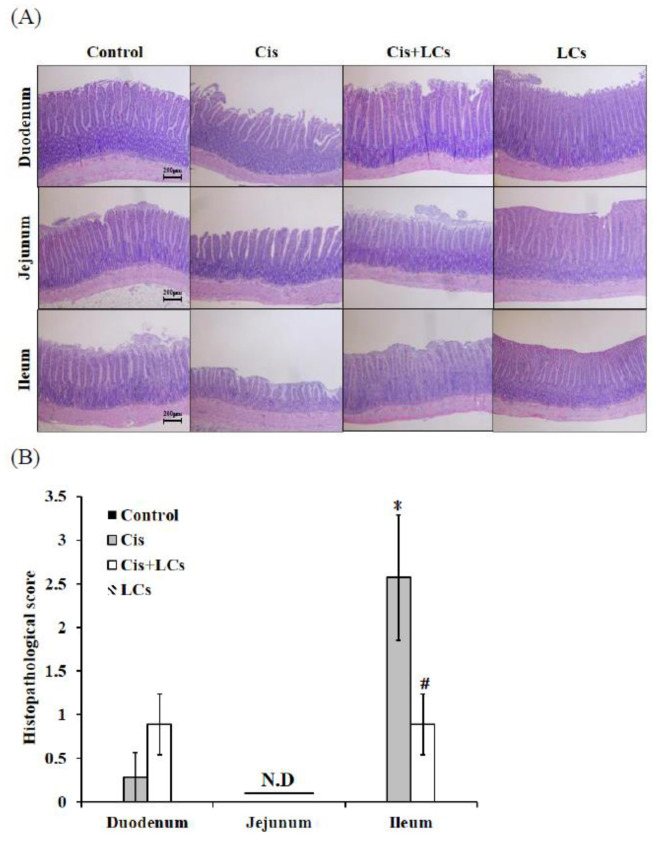

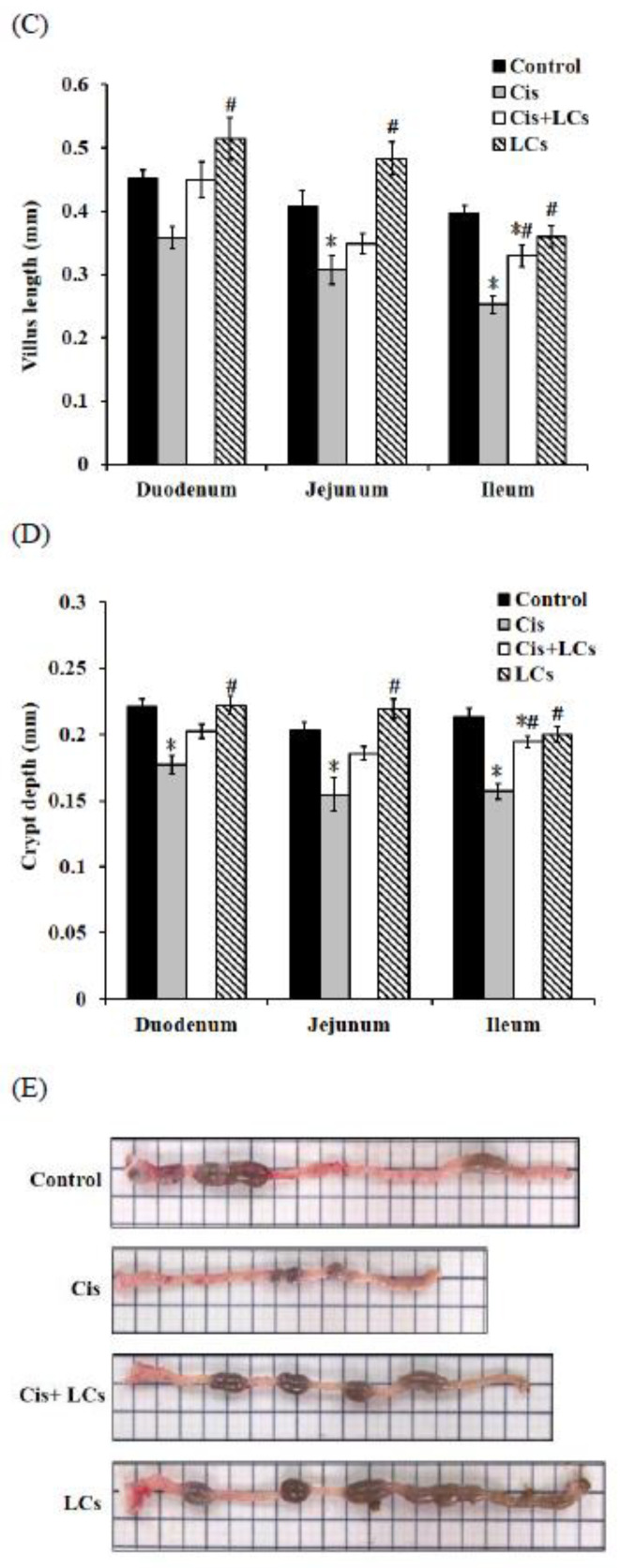

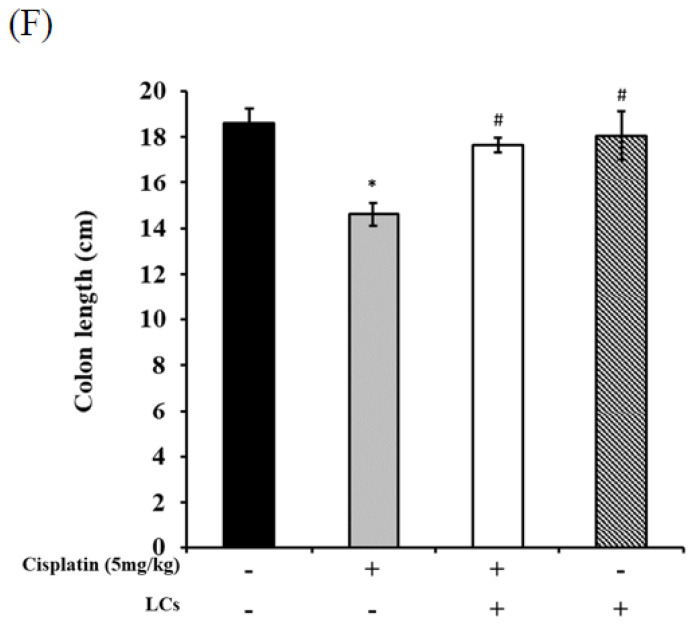
LCs reduces cisplatin-induced damage to intestinal tissue. (**A**) Representative images of H&E-stained duodenum, jejunum, and ileum sections. (**B**) Grading score of intestinal tissue damage. (**C**) Villus length. (**D**) Crypt depth. (**E**) Representative gross appearance of the colon. (**F**) Colon length. Results are shown as mean ± SEM (*n* = 7 per group). * *p* < 0.05 compared with the control group; # *p* < 0.05 compared with the cisplatin group.

Intestinal tissues (duodenum, jejunum, and ileum) were examined for histopathology by H&E to observe whether LCs have protective effects against cisplatin-induced mucositis. The normal histology of the small intestine in the control group was characterized by villi which were finger-like and intact, with intact crypt architecture, regular epithelial cells, and mucosal integrity. By contrast, cisplatin caused significant histological changes in ileum segments, and this damage involved shortened villi, loss of crypt architecture, and a pronounced inflammatory cell infiltrate in the lamina propria (Figure 4A–D). Apparently, the H&E-stained section of the ileum from the LCs-pretreated group showed prominent improvement as compared with the cisplatin-treated rats. Research has shown that the length of the colon decreased with inflammation. Hence, colon length could be used as an indicator of inflammation [21]. As shown in Figure 4E,F, the length of the colon in the cisplatin group was significantly shorter than that in the control group, whereas the LCs-pretreated group significantly prevented the decrease in the colon length as compared with the cisplatin group (*p* < 0.05). In addition, no significant differences in colon length were observed between the LCs alone group and control group.

### 4.8. LCs Alleviates Goblet Cell Damage and Gut-Derived Endogenous Endotoxin Production and Improves Intestinal Permeability in Rats Treated with Cisplatin

**Figure 5:**
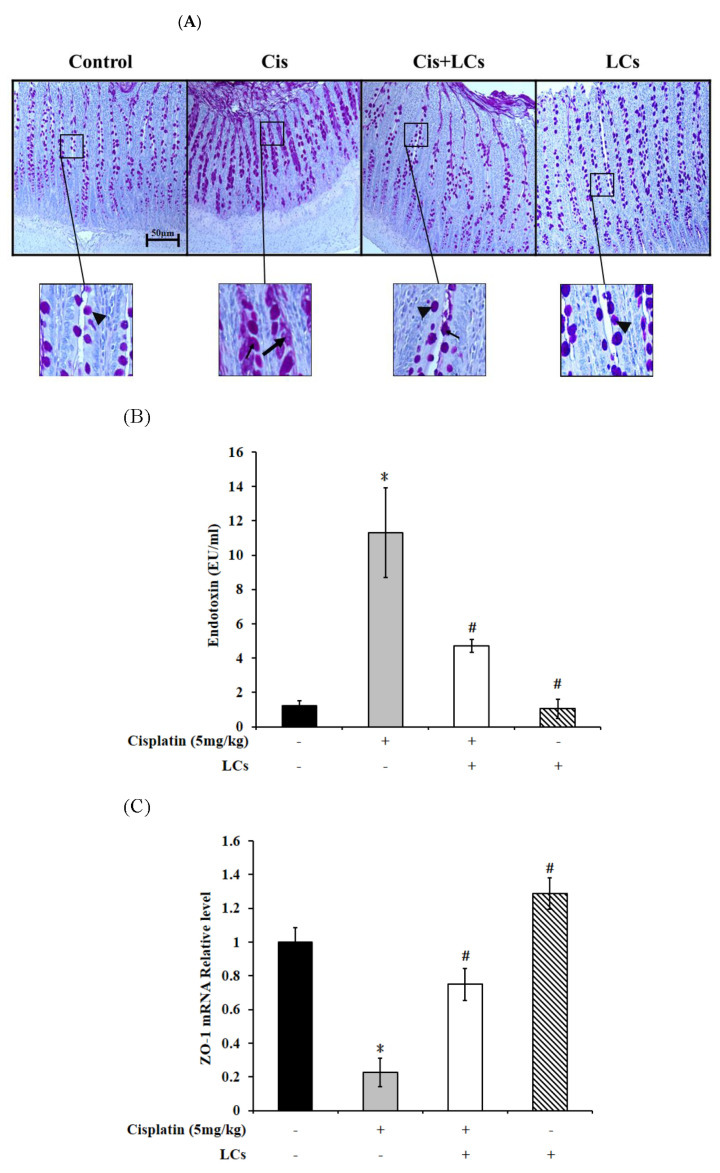
Effect of LCs supplementation on the integrity of goblet cells, tight junction protein expression, and endotoxin concentration in cisplatin-induced nephrotoxicity rats. (**A**) Goblet cell PAS staining of the ileum for microscopic histopathology was performed. Mucin-filled goblet cell (arrowheads); goblet cells were cavitated, and most mucous granules were depleted (triangle). (**B**) Serum endotoxin levels. (**C**) Expression of ZO-1 was subjected to real-time PCR analysis in the intestinal tissues. Results are shown as mean ± SEM (*n* = 3–7 per group). * *p* < 0.05 compared with the control group; # *p* < 0.05 compared with cisplatin group.

Furthermore, we observed that in the control and LCs alone treatment groups, most villus goblet cells were normal integrated goblet cells with a round-to-oval shape. The cytoplasm was filled with PAS-positive mucous granules. By contrast, rats treated with cisplatin showed that intervillous spaces contained a large amount of discharged mucins. Cisplatin treatment caused depletion of mucous granules from many villus goblet cells, which was a sign of cavitation. In rats pretreated with LCs prior to cisplatin treatment, the number of cavitated goblet cells decreased, and the discharged mucins decreased (Figure 5A).

As shown in Figure 5B, the concentration of serum endotoxin in the cisplatin group was significantly elevated compared with the control group, which decreased with LCs supplementation (*p* < 0.05). Gut permeability was evaluated by TJ proteins ZO-1. The results showed that the intestinal ZO-1 mRNA expression of cisplatin-treated rats significantly decreased compared with the control rats, but the decrease was markedly recovered by LCs supplementation (Figure 5C; *p* < 0.05). No significant difference in serum endotoxin levels and ZO-1 mRNA expression was observed between the LCs alone treatment group and control group.

### 4.9. Effect of LCs on Intestinal Digestive Enzyme Activity in Cisplatin-Treated Rats

**Figure 6:**
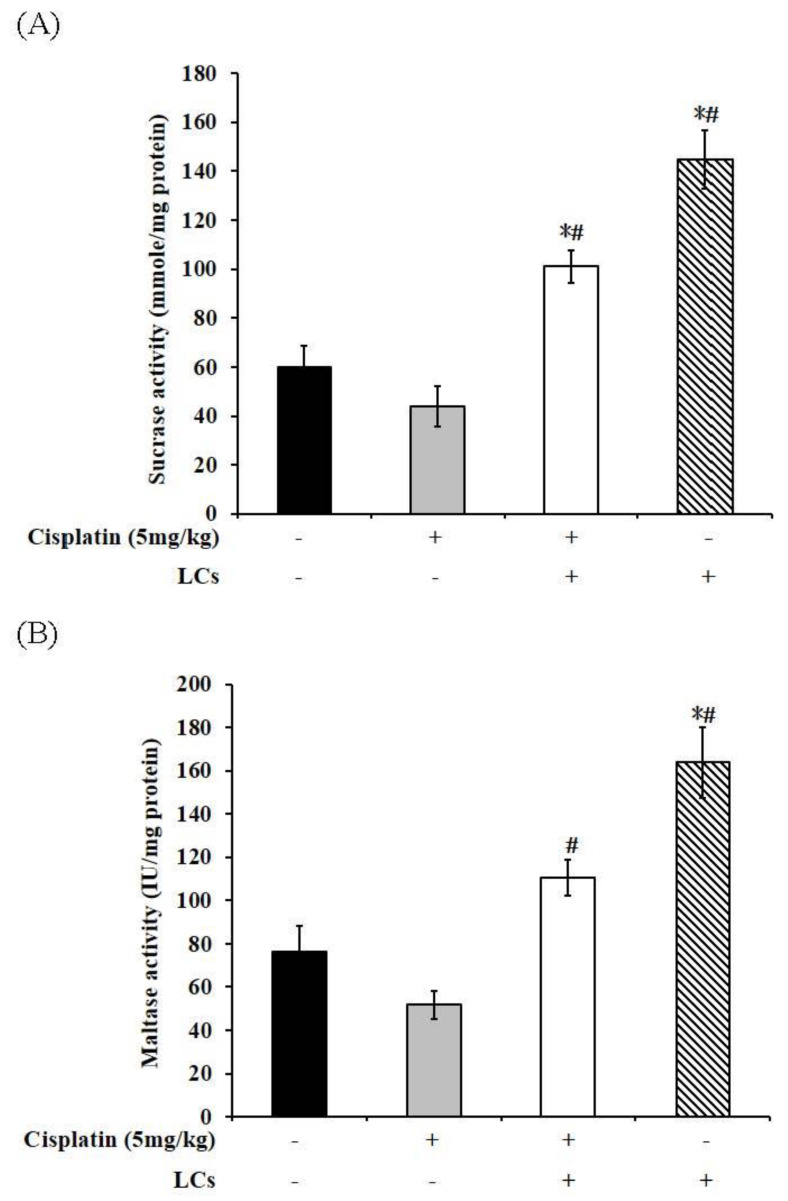

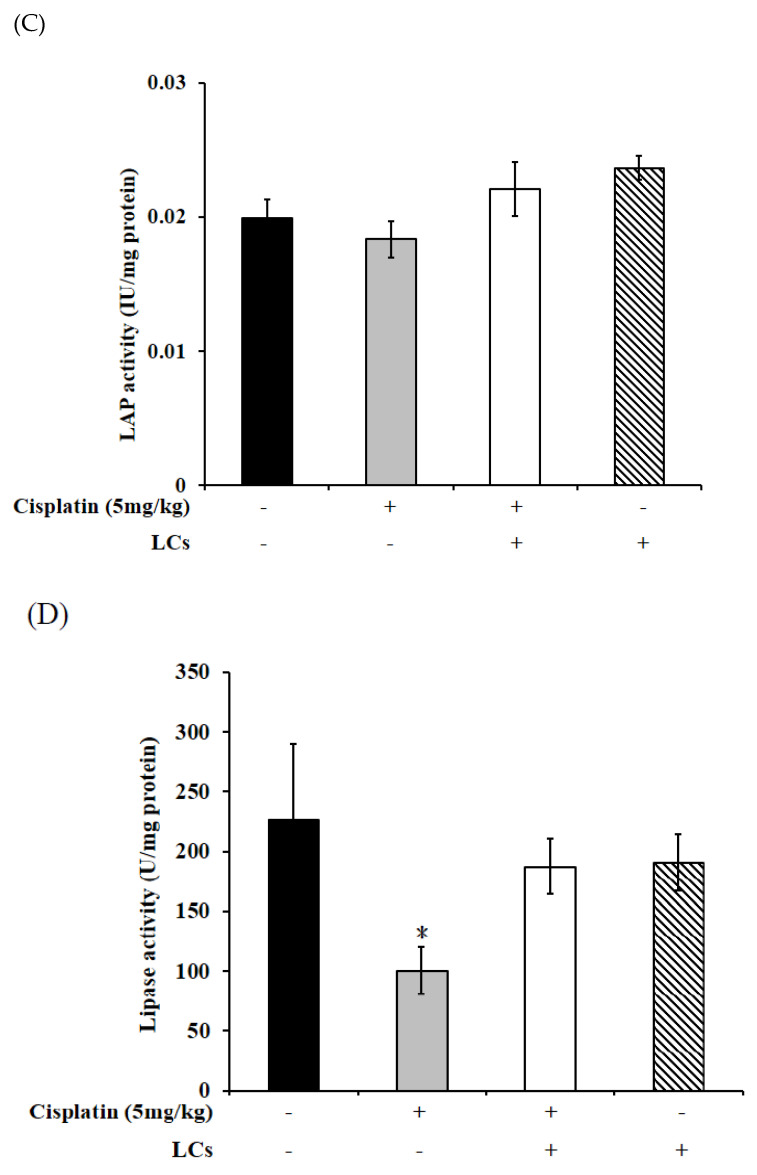
Digestive enzyme activity of cisplatin-treated rats supplemented with LCs. (**A**) Sucrase. (**B**) Maltase. (**C**) LAP. (**D**) Lipase. Results are shown as mean ± SEM (*n* = 7 per group). * *p* < 0.05 compared with the control group; # *p* < 0.05 compared with the cisplatin group.

The mucosal integrity was impaired, and the activities of intestinal digestive enzymes in the BBM (such as sucrase, maltase, leucine amino enzyme, and lipase) significantly decreased in rats treated with cisplatin, which led to a significant reduction in intestinal function [33]. The activity of the intestinal digestive enzymes was measured to evaluate digestive function. We observed that cisplatin l reduced lipase activity compared with the control group (Figure 6D), whereas supplementation of LCs in cisplatin-treated rats significantly improved the activities of sucrase and maltase as compared with cisplatin-treated rats (*p* < 0.05; Figure 6A,B). On the contrary, the activities of sucrase and maltase were markedly increased in rats fed with LCs as compared with the normal rats. However, LAP digestive enzyme activity did not show any significant difference among the four groups (Figure 6C).

### 4.10. LCs Administration Promotes Butyrate Production after Cisplatin Treatment

**Figure 7:**
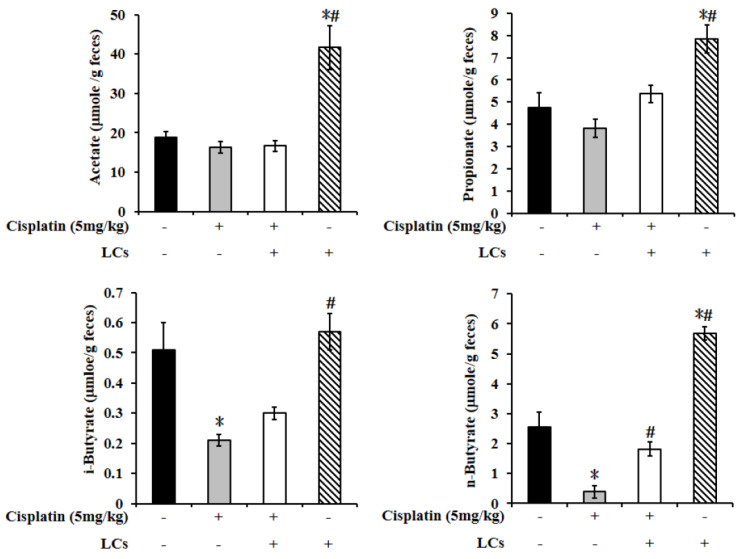
LCs probiotic restored butyrate production in cisplatin-induced nephrotoxicity rats. Results are shown as mean ± SEM (*n* = 7 per group). * *p* < 0.05 compared with the control group; # *p* < 0.05 compared with the cisplatin group.

We further investigated whether LCs’s protection could involve modulation of the SCFAs. In fecal samples, the levels of n-butyrate significantly declined in cisplatin-treated rats compared with the control group, and such levels were restored by LCs supplementation. In addition, among the four groups, the levels of acetate, propionate, and n-butyrate were highest in the LCs alone treatment group (Figure 7).

### 4.11. Restructuring of Gut Microbial Communities by LCs Intervention Prevents Cisplatin-Induced Dysbiosis

**Figure 8:**
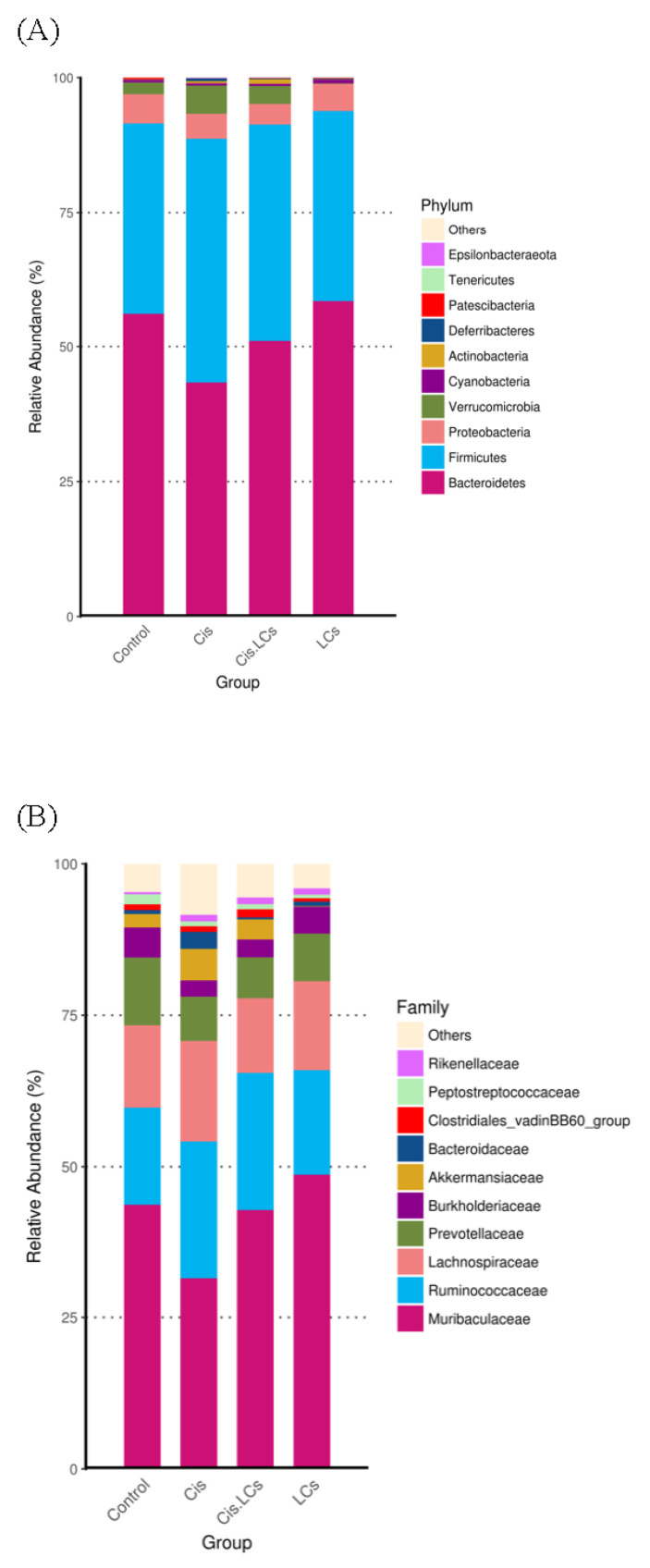

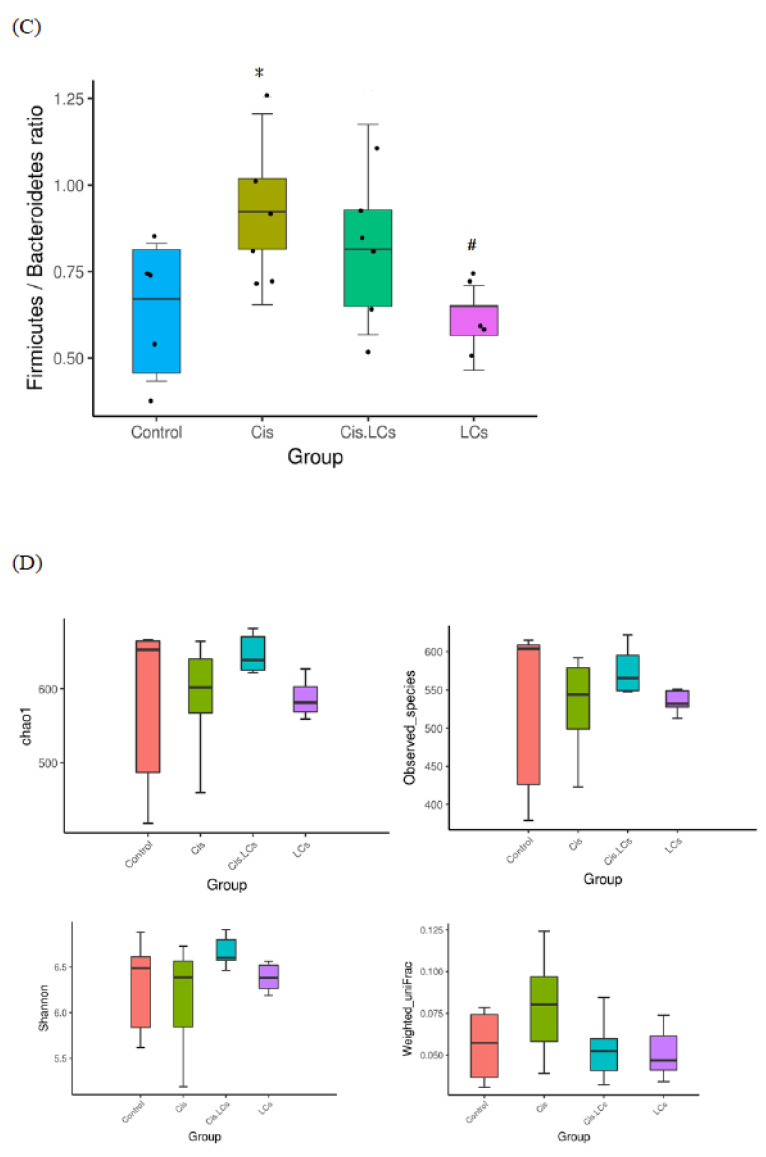

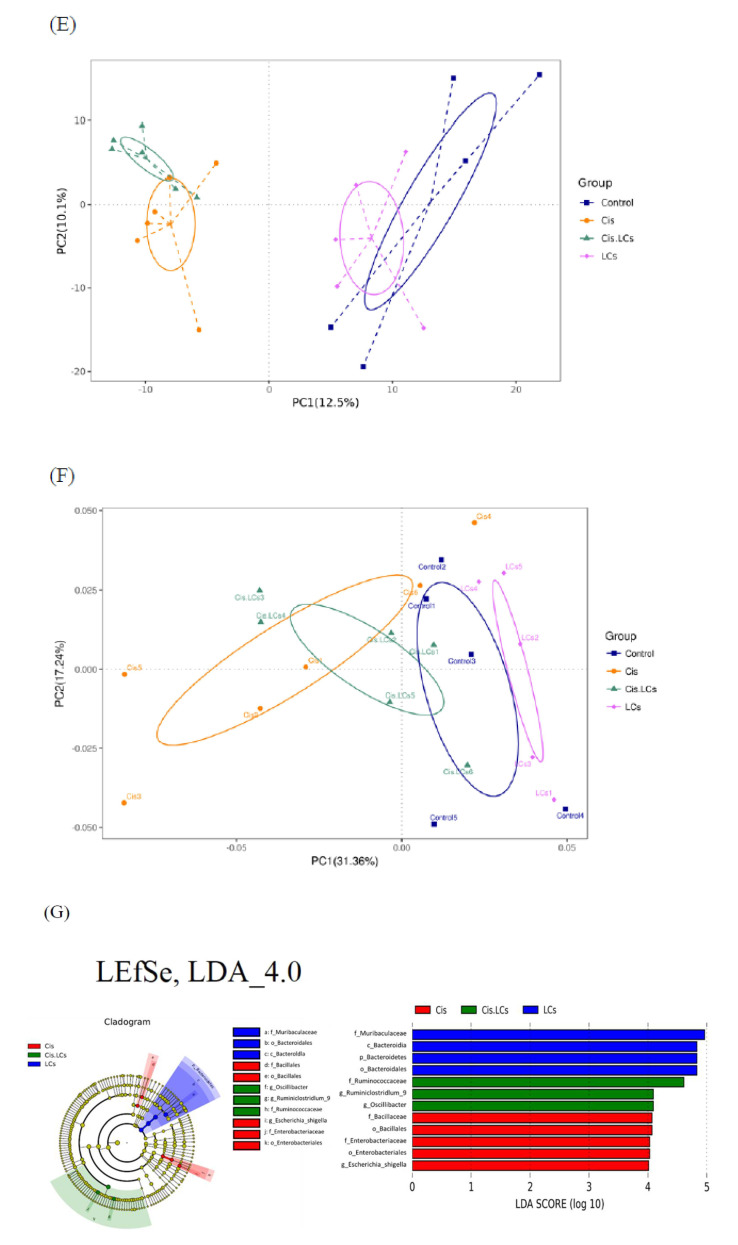

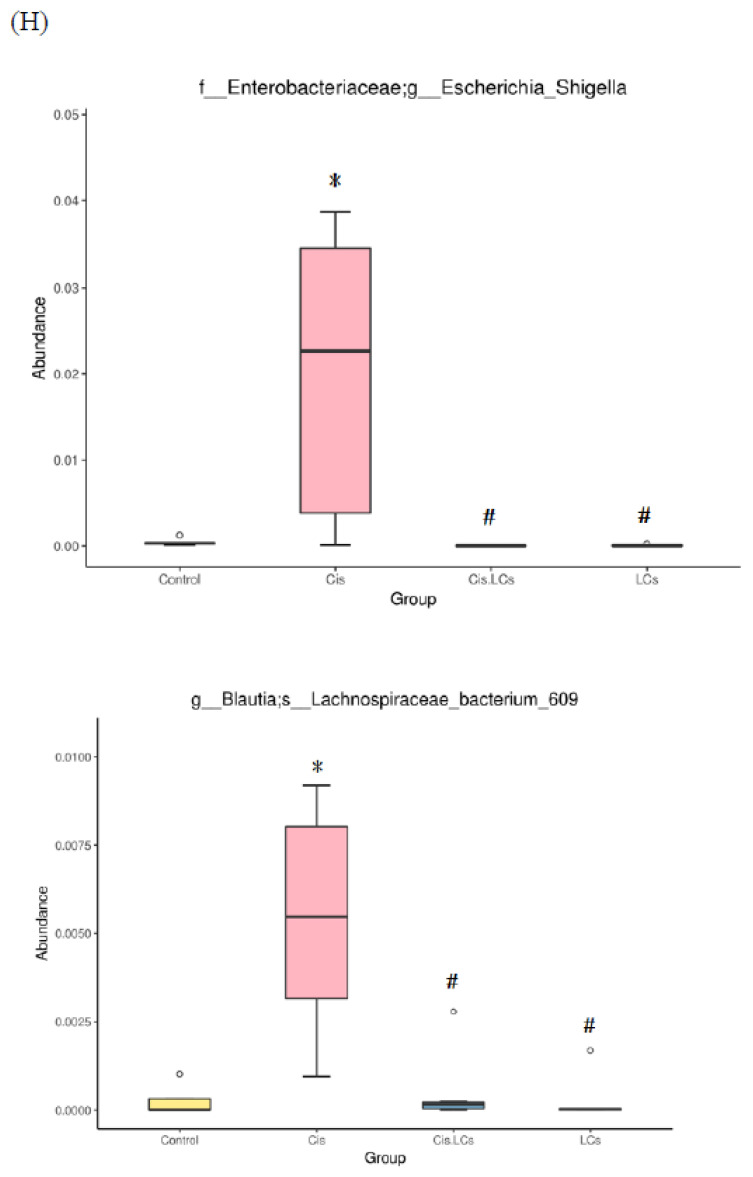

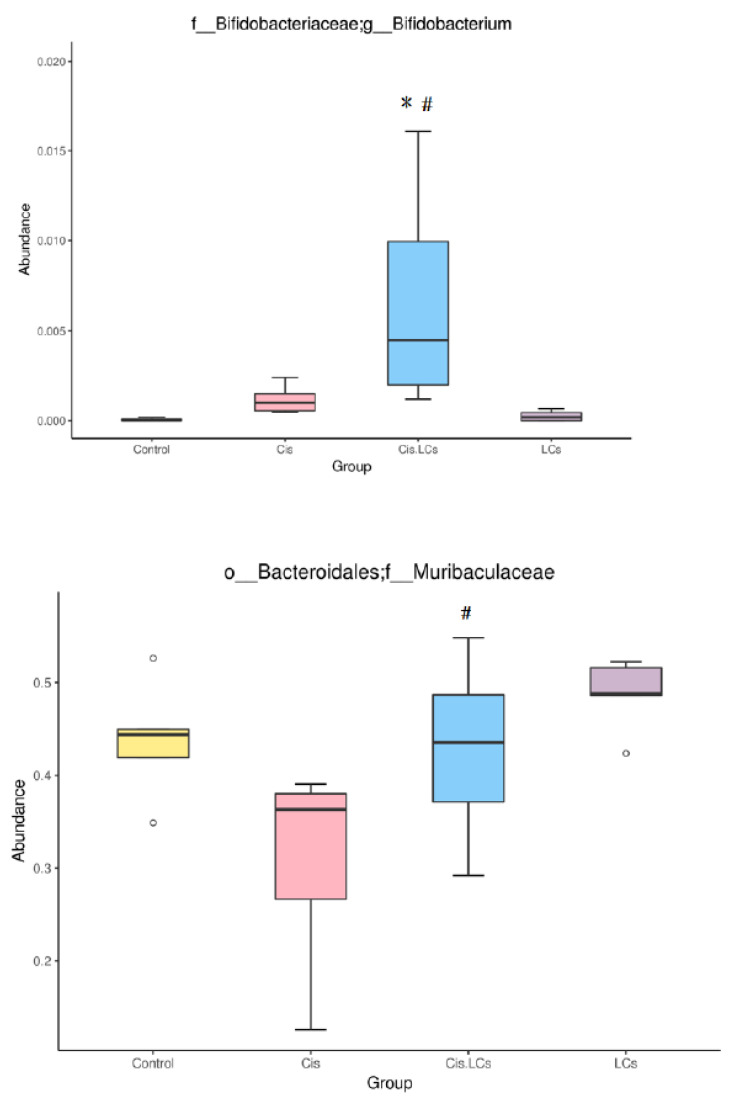

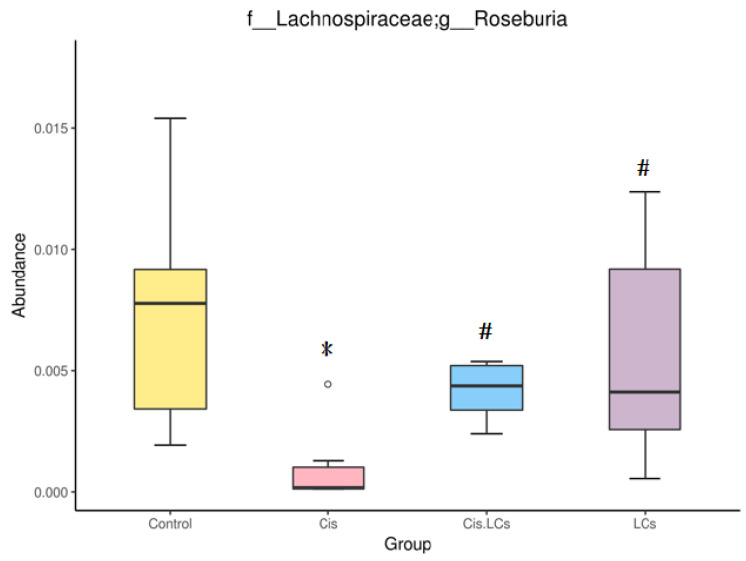
LCs probiotic restored the cisplatin-induced gut microbial dysbiosis. (**A**) Relative abundance of phylum. (**B**) Relative abundance of family. (**C**) Firmicutes/Bacteroidetes ratio. (**D**) Alpha diversity (Ace index, Chao index, Shannon index, and Simpson index) and Beta diversity (weighted UniFrac and unweighted Unifrac). (**E**) PCA. (**F**) PCoA analysis. (**G**) LDA score of LEfSe cladogram analysis. Indicator of bacterial groups with LDA score ≥ 4 in experimental groups. (**H**) Significant differences in microbial abundances according to taxa among the control, cisplatin, Cis+LCs, and LCs groups. Significant differences in microbial abundances according to taxa among the control, cisplatin, Cis+LCs, and LCs groups. * *p* < 0.05 compared with the control group; # *p* < 0.05 compared with the cisplatin group.

Evidence shows that cisplatin can cause a severe compositional and functional disturbance in the gut microbial community [1,2]. Next, NGS-16S analysis in feces of cecum was performed to investigate whether the reconstruction of gut microbiota induced by “good” bacteria could prevent kidney damage after cisplatin administration. At the phylum level (Figure 8A), compared with the control group, a lower abundance of *Bacteroidetes* and a higher abundance of *Firmicutes* were observed in cisplatin-treated rats, which resulted in an increased *Firmicutes*/*Bacteroidetes* (F/B) ratio in the cisplatin treatment group (Figure 8C). However, the cisplatin and LCs combined therapy did not significantly affect the F/B ratio. The richness, diversity, and evenness of gut microbiota were expressed by Chao1 and Shannon indices among the four groups (Figure 8D). The values of Chao1 and Shannon in the cisplatin group were lower than those of the control group, whereas higher values in alpha diversity indices were observed in the Cis+LCs group, compared with the cisplatin group. The Chao1 and Shannon indices were lowest in the LCs alone treatment group. These findings suggested that LCs intervention could reverse the ecological diversity and richness of gut microbiota in cisplatin-treated rats. Weighted UniFrac distance approach was used to compare the similarity of the gut microbial communities (β diversity) and the global composition of the microbiota among the groups. Figure 8C also shows the average weighted UniFrac distances. The smaller the value, the smaller the difference in species diversity among the samples.

The average weighted UniFrac distances were decreased by LCs administration in cisplatin-treated rats, indicating that the microbiota of Cis+LCs rats were more similar to one another when compared with cisplatin-treated rats.

The Principal Component Analysis (PCA) result of principal component analysis based on the OTU level is shown in Figure 8E, and all samples in the four groups can be clearly distinguished. The cisplatin and Cis+LCs groups can be clearly separated, indicating that the intervention of LCs significantly altered the composition of microbial communities in cisplatin-treated rats. We also found that the composition of microbial communities between the control group and LCs alone group was similar.

The PCoA plots showed that the closer the distance among the samples, the more similar the species composition structure; therefore, these microbiota will cluster together. As shown in Figure 8F, PCoA plots revealed that the rats fed with LCs alone had a similar gut bacterial composition with small intra-group variation, but the rats treated with cisplatin and co-treated with LCs exhibited greater intra-group variations.

The LEfSe algorithm was used to identify microbes as potential biomarkers at multiple taxonomical levels, which characterized the differences among these four groups within the digestive tract, and to identify taxonomic differences between cisplatin-treated and LCs-supplemented rats. All potential biomarkers (LDA score > 4) are shown in Figure 8G. At the family level, the cisplatin-treated rats showed increased *Enterobacteriaceae* and *Bacillaceae*, whereas *Ruminococcaceae* was enriched in LCs-supplemented rats. At the genus level, *Escherichia-Shigella* was the dominant bacteria in cisplatin-treated rats, whereas the biomarkers in cisplatin-treated rats fed with LCs were *Ruminiciostridium_9* and *Oscillibacter*. These data demonstrated that cisplatin increased the relative abundance of pathogenic bacteria, such as *Escherichia-Shigella* and *Enterobacteriaceae*, which was thoroughly reversed by LCs supplementation. Notably, the dominant bacteria in LCs alone treatment group were primarily *Muribaculaceae*.

We used metagenomeSeq to identify taxa that showed statistically significant genera or species levels at different treatment groups (Figure 8H). *Escherichia-Shigella* and *Lachnospiraceae bacterium 609* were highest within the gastrointestinal tract of rats treated with cisplatin compared with other treatment groups, and this increase was reversed by the supplementation of LCs. By contrast, we observed that cisplatin reduced the amount of *Muribaculaceae* and *Roseburia* compared with the control group, whereas supplementation of LCs in cisplatin-treated rats significantly increased both bacteria. Notably, the amounts of *Bifidobacterium* in the Cis+LCs group were highest among the four groups.

## 5. Discussion

Accumulating evidence indicates that cisplatin-induced nephrotoxicity is associated with the strong imbalance of common gut microbes (dysbiosis) [2,3], whereas probiotics have an important protective effect on the pathogenesis of cisplatin-induced nephrotoxicity [2], which is associated with possible changes to the composition of the commensal bacteria. However, mechanistic insights are lacking, particularly the mechanism of probiotics and probiotic-derived components, such as metabolites, in attenuating cisplatin-induced intestinal mucositis and kidney damage. 

MPO is a marker of neutrophils, while F4/80 is the surface antigen of macrophages, and it has been considered as a macrophage infiltration marker [34]. A large number of neutrophils and macrophage infiltration in renal tissues could result in the deterioration of renal tissues after cisplatin administration [31,35]. In the present study, we observed that the protective effects of LCs on cisplatin-induced nephrotoxicity were mediated by blocking neutrophil and macrophage infiltration into the damaged kidneys. The expression of KIM-1 in proximal tubule cells in human kidney biopsy sections from patients with acute tubular necrosis was extensive, and it may serve as a novel biomarker for renal proximal tubule injury [36]. A previous study reported that cisplatin markedly elevated renal tubular expressions of KIM-1 [37] and LCs treatment reduced the significant upregulation of renal KIM-1, suggesting that LCs decreased renal tubular injury. These results confirmed that the possible beneficial effects of *L. reuteri* and CBM 588 were associated with their diverse bioactivities, including anti-inflammatory and antioxidative effects and other potential biological properties [16,21,38].

Multiple low-dose injections of cisplatin can cause renal interstitial fibrosis, which is associated with ROS accumulation and inflammatory response in tubular cells [39]. Collagen consists of hydroxyproline or proline, glycine, and an additional amino acid, whereas hydroxyproline is found in collagen of renal origin. A previous study has proposed that the increase in 4-hydroxyproline caused by cisplatin may be due to increased extracellular matrix degradation of kidney epithelial cells [40]. The breakdown of collagen results in increased hydroxyproline levels in the kidney, which is associated with the development of fibrosis [32]. MIYAIRI 588 may provide a new strategy for antifibrotic therapy, as evidenced by the reduced hepatic protein levels of fibrosis-related factors, including α-SMA and collagen I, and hepatic collagen deposition [41]. Our studies revealed that renal fibrosis was markedly alleviated in rats pretreated with LCs, as evidenced by the reduced expression of α-SMA, percentage areas of collagen staining, and hydroxyproline levels in kidney tissues. These results match the histological findings, suggesting that LCs has a good recovery effect on fibrosis. In addition, caspase-3 may be involved in renal apoptosis and subsequent renal fibrosis [42]. Our results are consistent with the published results, and specific probiotic mix pretreatment can reduce cisplatin-induced kidney apoptosis and inflammation [19]. Therefore, probiotics were considered as an effective chemoprotectant, which attenuates cisplatin-induced nephrotoxicity [2]. 

The disruption of the intestinal barrier is associated with the elevated LPS level [43]. When gut permeability is increased, the harmful bacteria or endotoxins will translocate from the gut into circulation and trigger the systemic inflammatory response. The previous results showed that chemotherapy-associated gastrointestinal toxicity is associated with increased relative abundance of LPS-producing bacteria [44]. Probiotics could inhibit survival and colonization of enteric pathogenic bacteria through some mechanisms, including competitive exclusion, production of antimicrobial metabolites, and modulation of the composition of the intestinal microbiota [45]. Studies have shown that *L. reuteri* can activate macrophage phagocytic activity to kill intracellular pathogens and reduce the colonization of pathogenic bacteria in the liver and spleen [38]. Some authors speculate that *L. reuteri* ameliorates the inflammatory response by remodeling the gut microbiota [46] and exerts antibacterial activity by secreting bacteriocins, such as reuterin [38]. Reuterin, an intermediate compound produced by some strains of *L. reuteri*, is an antimicrobial substance that can widely inhibit the growth of Gram-positive and Gram-negative pathogenic bacteria. *L. reuteri* FN041 alleviates the disruption of the intestinal epithelial barrier and intestinal dysbiosis, reduces serum endotoxin levels, and increases SCFA production in high-fat diet-induced metabolic disorder models [16]. 

On the other hand, CBM588 may ameliorate inflammation in the colon by decreasing the levels of pro-inflammatory cytokines [47] and increasing anti-inflammatory lipid metabolites [21]. CBM 588 also enhanced butyric acid production, which protects the mucin layer covering the intestinal epithelia and significantly reduces epithelial damage [48]. The main SCFAs are acetate, propionate, and butyrate. SCFAs have potential beneficial effects; in particular, butyrate exerts anti-inflammatory [10,49], nephroprotective effects [50,51], modifies the microbial community [43], and regulates the mucosal immune response [52]. Butyrate could also increase SCFA-producing bacteria and decrease pathogenic bacteria, such as endotoxin-secreting bacteria [49] and endotoxin production [43]. Our results demonstrated that LCs intervention promoted butyrate production, which protects epithelial barrier function and reduces the risk of intestinal inflammation [51].

Goblet cells are the first line of defense of the mucosal surface, and they secrete mucins that form a protective gel-like covering in the gastrointestinal lumen. Therefore, mucins serve as a protective layer. The loss of the mucus layer caused by inflammation triggers the release of goblet cell mucus. LPS could cause endotoxemia, which results in systemic inflammation, depletion of mucous granules from goblet cells, and decreases the intracellular storage of goblet cell mucins in the mucosal villi epithelium [53]. We speculate that cisplatin can cause the disintegration of goblet cells, which is associated with LPS production. LCs pretreatment showed protection against cisplatin-induced goblet cell disintegration, thereby preserving goblet cell mucins in the mucosal villi epithelium. 

The intestinal epithelial cells are tightly bound together by intercellular junctional complexes, which regulate the paracellular permeability, and they are crucial for the integrity of the epithelial barrier. Tight junctions (TJ) are multifunctional protein complexes that maintain epithelial barrier integrity. Proteins including occludin, ZO-1, and claudin-1 are important components of intestinal tight junctions. Cisplatin-induced oxidative stress and inflammation [5] lead to increased intestinal permeability [54], which is associated with the downregulation of TJ proteins such as occludin and ZO-1 [2]. Moreover, cisplatin-driven dysbiosis can lead to excessive secretion of uremic toxins, which may damage renal tubular cells and can impair the intestinal barrier function [2]. Indole is a direct precursor of the uremic toxin IS. IS, a potential gut-derived toxin, is primarily produced through tryptophan metabolism by undesirable bacteria. The increase in serum IS levels could be associated with an increase in the abundance of specific microbiota [55] and may worsen the progression of chronic kidney disease (CKD) [2,56]. A previous study has indicated that *L. reuteri* strains treatment can restore mucosal barrier function by via enhancing antioxidant activities and TJ-associated protein occludin and claudin-3 expression [23]. Furthermore, CBM 588 significantly reduces serum endotoxin levels and hepatic inflammatory indexes and dramatically enhances TJ protein expression (ZO-1 and occludin) in rats with nonalcoholic fatty liver disease [41]. The increase in serum levels of endotoxin (Figure 5B) might be associated with the increase in some potentially pathogenic species abundance, such as Bacteroides species [56,57]. *Shigella* is a Gram-negative genus of bacteria, and it is an important source of endotoxin. *Escherichia-Shigella* (β-glucuronidase-producing bacteria) belongs to secondary bile acid-producing bacteria [30] and could induce inflammatory response and increase intestinal permeability [44]. Notably, *Escherichia-Shigella* spp. is highly associated with the levels of IS in CKD patients [6]. Moreover, *Enterobacteriaceae*, which produce indole or p-cresol, are higher in subjects with uremia compared with healthy subjects [55]. Patients with hepatic fibrosis had a higher abundance of the *Escherichia-Shigella* and *Enterobacteriaceae* families [58]. Additionally, CKD patients showed higher levels of *Enterobacteriaceae* but lower levels of *Ruminococcaceae* compared with the healthy individuals [59]. Consistent with previous research, our results showed that cisplatin disturbed the intestinal microbiota that exacerbated mucosal damage [1] and kidney damage [2,19] and induced *Escherichia-Shigella* as the dominant genera [60]. 

Our study found that LCs can change the composition of the gut microbiome and shift the gut microbial community toward certain beneficial bacteria, including *Bifidobacterium* and *Oscillibacter*. CBM 588 was used in our study and increased *Bifidobacterium* abundance, carbohydrate metabolite production, and SCFA production in an antibiotic-induced colitis model, thereby improving colon epithelial damages [21]. *Bifidobacterium* and *Bacteroidales* family *S24-7* belong to potential fiber-degrading bacteria, which could efficiently ferment fiber into butyrate [61]. The abundance of *Bifidobacterium* is correlated with the mucus growth rate [61]. *Bifidobacteria* could reduce serum levels of IS through modulating the intestinal microflora in hemodialysis patients [62]. In addition, an increase in the relative abundances of *Bifidobacterium* in the GI tract reduces intestinal endotoxin production and maintains barrier function [63]. Notably, in the current study, *Muribaculaceae* was enriched within the gastrointestinal tracts of rats receiving cisplatin plus LCs as compared with cisplatin-treated rats and was dominant in rats treated with LCs alone (Figure 8G,H). *Muribaculaceae*, also called S24-7, is involved in complex carbohydrate degradation, and it plays an immunomodulatory role [64]. *Muribaculaceae* is a butyrate-producing bacterium which is positively correlated with immune responses, including natural killer cell and NF-κB signaling [65]. 

Butyrate-producing bacteria (*Roseburia*, *Coprococcus*, *Bifidobacterium*, *Ruminococcus*, and *Faecalibacterium*) were depleted after chemotherapy, which may also reduce the production of SCFAs [63]. However, probiotics could enhance the abundance of SCFAs-producing bacteria [3]. With the increase of the butyrate levels after LCs intervention in our study, some butyrate-producing bacteria, such as *Bifidobacterium*, *Ruminiclostridium_ 9*, and *Ruminococcaceae*, were also enriched. *Ruminococcaceae* was increased after *C. butyricum* treatment [13]. An increase in the relative abundance of *Ruminococcaceae*, *Bacteroidales_S24-7_group*, *Oscillibacter*, and *Akkermansia* may relieve HFD-induced inflammation and metabolic syndrome [57]. Hence, supplementation of LCs improves cisplatin-induced intestinal damage, which could be associated with the enhancement of the relative abundance of *Ruminococcaceae* and a lower abundance of *Enterobacteriaceae* [57]. Moreover, butyrate-producing bacteria may alleviate intestinal permeability and maintain barrier function [63]. 

Our results also showed that oral supplementation of LCs increased the abundance of *Oscillibacter* (Figure 8G) and *Roseburia* (Figure 8H) after cisplatin treatment. A previous study showed that *Oscillibacter* abundance was increased in rats with a high-fat diet. The increased *Oscillibacter* abundance is associated with the metabolic syndrome and development of obesity-related metabolic disorders [57]. However, another clinical trial revealed that *Oscillibacter* and *Lachnospiraceae* were significantly reduced in patients with amyotrophic lateral sclerosis compared with the healthy controls [66]. Moreover, *Oscillibacter* was considered as a producer of anti-inflammatory metabolites, which could reduce Th17 polarization and promote the differentiation of anti-inflammatory Treg/Tr1 cells in the gut [3]. Therefore, the roles of *Oscillibacter* involved in the physiology of the host need further investigation. *Roseburia* spp. (butyrate-producing bacteria) belong to the *Firmicutes* phylum and exhibit anti-inflammatory effects on patients with ulcerative colitis [67]. In patients with CKD, impaired kidney function was positively correlated with the levels of *Enterobacteriaceae* and *E. coli* but negatively correlated with the levels of *Bifidobacterium* spp. and *Roseburia* spp. [68], indicating that the depletion of these two butyrate-producing bacteria may contribute to CKD-associated inflammation and CKD progression.

Taken together, these findings demonstrate that the LCs probiotics increase butyrate production and butyrate-producing bacteria, which exert a broad range of physiological effects on the host, including the use of butyrate as fuel for intestinal cells, maintaining mucosal integrity, and alleviating intestinal inflammation. This was associated with a significant decrease in circulating LPS, and showed a significant alleviation of renal damage [51]. Butyrate can not only inhibit pathogenic bacteria while stimulating the growth of beneficial bacteria [69], but can also decrease the levels of microbiota-dependent components, such as endotoxin [49]. Another reason for LCs ameliorated cisplatin nephrotoxicity was probably because of a decrease in the number of indole-producing bacteria and maintenance of intestinal barrier function caused by the decreased levels of the IS [56] and endotoxin [41] or the elevation in the amount of *Bifidobacterium* [62,63]. We observed the relationship between the gut–metabolite–kidney axis and the pathogenesis of renal impairment [6] and identified the protective role of probiotics as a mediator of the gut–kidney axis in cisplatin-induced nephrotoxicity.

## 6. Conclusions

Cisplatin caused intestinal microflora dysbiosis, which might change the microbiota-derived metabolites and affect the gut–kidney axis, leading to nephrotoxicity. Some evidence in this study was proposed to explain the potential mechanisms of LCs in the prevention of cisplatin-induced side effects. These potential mechanisms included changes in the structure of intestinal commensal bacteria, such as increasing butyrate-producing bacteria, decreasing pathogenic bacteria, and modulating the levels of microbiota-dependent metabolites and components, such as SCFAs, uremic toxins, and endotoxin. In addition, our study demonstrated that pre-administration of LCs had a protective effect on cisplatin-induced renal toxicity through inhibiting apoptosis, oxidative stress, and inflammation in renal tissues.

## Figures and Tables

**Table 1:** Blood biochemical variables.

Group	Control	Cisplatin	Cis+LCs	LCs
AST (U/L)	0.121 ± 6.38	109.14 ± 6.25	0101.89 ± 6.55	104.29 ± 6.73
ALT (U/L)	00.53 ± 2.50	0.49 ± 6.26	034.89 ± 2.45 *^#^	038.14 ± 1.62 *
BUN (mg/dL)	17.26 ± 1.18	119.61 ± 10.94 *	0056.6 ± 8.30 *^#^	015.73 ± 1.32 ^#^
Creatinine (mg/dL)	01.33 ± 0.06	02.87 ± 0.32 *	001.99 ± 0.09 *^#^	001.15 ± 0.06 ^#^
Cystatin C (mg/L)	01.27 ± 0.05	03.99 ± 0.44 *	.002.06 ± 0.19 ^#^	001.04 ± 0.02 ^#^
UA (mg/dL)	04.00 ± 0.39	01.37 ± 0.17 *	02.66 ± 0.16 *^#^	004.03 ± 0.26 ^#^
IgA (mg/dL)	00.90 ± 3.78	281.29 ± 27.25 *	152.89 ± 13.12 *^#^	076.43 ± 2.19 ^#^
Indoxyl Sulfate (mg/mL)	00.91 ± 0.02	02.76 ± 0.21 *	01.61 ± 0.03 *^#^	000.86 ± 0.01 ^#^

Results are shown as mean ± SEM (*n* = 7 per group). * *p* < 0.05 compared with the control group; ^#^ *p* < 0.05 compared with the cisplatin group.

## Data Availability

The data sets used and/or analyzed during the current study are available from the corresponding author on reasonable request.

## Supplementary Materials

The following are available online at https://www.mdpi.com/article/10.3390/nu13082792/s1, Figure S1: Effects of cisplatin and LCs supplementation on digestive function. (A) Body weight changes during the experimental period. (B) Food intake changes during the experimental period. (C) Feeding efficiency ratio. (D) Gastric content. (E) Gastric emptying index, Figure S2: Effects of cisplatin and LCs supplementation on antioxidant/oxidant status in kidney tissues. (A) MDA. (B) GPx activity. (C) Catalase, Table S1: Effect of cisplatin and probiotics on organ weights and relative tissue weights, Table S2: The pH, moisture content, wet and dry weights of stool after the treatment of cisplatin and probiotics.

## References

[1] Angelakis E. (2017). Weight gain by gut microbiota manipulation in productive animals. Microb. Pathog..

[2] Ansari M.A. (2017). Sinapic acid modulates Nrf2/HO-1 signaling pathway in cisplatin-induced nephrotoxicity in rats. Biomed. Pharmacother..

[3] Arivarasu N.A., Priyamvada S., Mahmood R. (2013). Oral administration of caffeic acid ameliorates the effect of cisplatin on brush border membrane enzymes and antioxidant system in rat intestine. Exp. Toxicol. Pathol..

[4] Chen D., Jin D., Huang S., Wu J., Xu M., Liu T., Dong W., Liu X., Wang S., Zhong W. (2020). Clostridium butyricum, a butyrate-producing probiotic, inhibits intestinal tumor development through modulating Wnt signaling and gut microbiota. Cancer Lett..

[5] Chen X., Wei W., Li Y., Huang J., Ci X. (2019). Hesperetin relieves cisplatin-induced acute kidney injury by mitigating oxidative stress, inflammation and apoptosis. Chem. Biol. Interact..

[6] Chen X., Xu J., Su Y., Zhu W. (2018). Effects of Intravenous Infusion with Sodium Butyrate on Colonic Microbiota, Intestinal Development- and Mucosal Immune-Related Gene Expression in Normal Growing Pigs. Front. Microbiol..

[7] Cui Y., Liu L., Dou X., Wang C., Zhang W., Gao K., Liu J., Wang H. (2017). Lactobacillus reuteri ZJ617 maintains intestinal integrity via regulating tight junction, autophagy and apoptosis in mice challenged with lipopolysaccharide. Oncotarget.

[8] Denk S., Weckbach S., Eisele P., Braun C.K., Wiegner R., Ohmann J.J., Wrba L., Hoenes F.M., Kellermann P., Radermacher P. (2018). Role of Hemorrhagic Shock in Experimental Polytrauma. Shock.

[9] Dicksved J., Schreiber O., Willing B., Petersson J., Rang S., Phillipson M., Holm L., Roos S. (2012). Lactobacillus reuteri maintains a functional mucosal barrier during DSS treatment despite mucus layer dysfunction. PLoS ONE.

[10] Endo H., Niioka M., Kobayashi N., Tanaka M., Watanabe T. (2013). Butyrate-producing probiotics reduce nonalcoholic fatty liver disease progression in rats: New insight into the probiotics for the gut-liver axis. PLoS ONE.

[11] Espandiari P., Rosenzweig B., Zhang J., Zhou Y., Schnackenberg L., Vaidya V.S., Goering P.L., Brown R.P., Bonventre J.V., Mahjoob K. (2010). Age-related differences in susceptibility to cisplatin-induced renal toxicity. J. Appl. Toxicol..

[12] Forsgard R.A., Marrachelli V.G., Korpela K., Frias R., Collado M.C., Korpela R., Monleon D., Spillmann T., Osterlund P. (2017). Chemotherapy-induced gastrointestinal toxicity is associated with changes in serum and urine metabolome and fecal microbiota in male Sprague-Dawley rats. Cancer Chemother. Pharmacol..

[13] Gonzalez A., Krieg R., Massey H.D., Carl D., Ghosh S., Gehr T.W.B., Ghosh S.S. (2019). Sodium butyrate ameliorates insulin resistance and renal failure in CKD rats by modulating intestinal permeability and mucin expression. Nephrol. Dial. Transplant.

[14] Grusovin M.G., Bossini S., Calza S., Cappa V., Garzetti G., Scotti E., Gherlone E.F., Mensi M. (2020). Clinical efficacy of Lactobacillus reuteri-containing lozenges in the supportive therapy of generalized periodontitis stage III and IV, grade C: 1-year results of a double-blind randomized placebo-controlled pilot study. Clin. Oral. Investig..

[15] Gryp T., Huys G.R.B., Joossens M., Van Biesen W., Glorieux G., Vaneechoutte M. (2020). Isolation and Quantification of Uremic Toxin Precursor-Generating Gut Bacteria in Chronic Kidney Disease Patients. Int. J. Mol. Sci..

[16] Hagihara M., Kuroki Y., Ariyoshi T., Higashi S., Fukuda K., Yamashita R., Matsumoto A., Mori T., Mimura K., Yamaguchi N. (2020). Clostridium butyricum Modulates the Microbiome to Protect Intestinal Barrier Function in Mice with Antibiotic-Induced Dysbiosis. iScience.

[17] Hagihara M., Yamashita R., Matsumoto A., Mori T., Kuroki Y., Kudo H., Oka K., Takahashi M., Nonogaki T., Yamagishi Y. (2018). The impact of Clostridium butyricum MIYAIRI 588 on the murine gut microbiome and colonic tissue. Anaerobe.

[18] Hayashi A., Sato T., Kamada N., Mikami Y., Matsuoka K., Hisamatsu T., Hibi T., Roers A., Yagita H., Ohteki T. (2013). A single strain of Clostridium butyricum induces intestinal IL-10-producing macrophages to suppress acute experimental colitis in mice. Cell Host Microbe.

[19] Huang K., Yan Y., Chen D., Zhao Y., Dong W., Zeng X., Cao Y. (2020). Ascorbic Acid Derivative 2-O-beta-d-Glucopyranosyl-l-Ascorbic Acid from the Fruit of Lycium barbarum Modulates Microbiota in the Small Intestine and Colon and Exerts an Immunomodulatory Effect on Cyclophosphamide-Treated BALB/c Mice. J. Agric. Food. Chem..

[20] Hwang S., Park J., Kim J., Jang H.R., Kwon G.Y., Huh W., Kim Y.G., Kim D.J., Oh H.Y., Lee J.E. (2017). Tissue expression of tubular injury markers is associated with renal function decline in diabetic nephropathy. J. Diabetes Complicat..

[21] Jiang P., Yang W., Jin Y., Huang H., Shi C., Jiang Y., Wang J., Kang Y., Wang C., Yang G. (2019). Lactobacillus reuteri protects mice against Salmonella typhimurium challenge by activating macrophages to produce nitric oxide. Microb. Pathog..

[22] Kim J.Y., Jo J., Kim K., An H.J., Gwon M.G., Gu H., Kim H.J., Yang A.Y., Kim S.W., Jeon E.J. (2019). Pharmacological Activation of Sirt1 Ameliorates Cisplatin-Induced Acute Kidney Injury by Suppressing Apoptosis, Oxidative Stress, and Inflammation in Mice. Antioxidants.

[23] Koppe L., Mafra D., Fouque D. (2015). Probiotics and chronic kidney disease. Kidney Int..

[24] Kuo C.H., Lee S.H., Chen K.M., Lii C.K., Liu C.T. (2011). Effect of garlic oil on neutrophil infiltration in the small intestine of endotoxin-injected rats and its association with levels of soluble and cellular adhesion molecules. J. Agric. Food Chem..

[25] Lakritz J.R., Poutahidis T., Levkovich T., Varian B.J., Ibrahim Y.M., Chatzigiagkos A., Mirabal S., Alm E.J., Erdman S.E. (2014). Beneficial bacteria stimulate host immune cells to counteract dietary and genetic predisposition to mammary cancer in mice. Int. J. Cancer.

[26] Lee T.H., Park D., Kim Y.J., Lee I., Kim S., Oh C.T., Kim J.Y., Yang J., Jo S.K. (2020). Lactobacillus salivarius BP121 prevents cisplatininduced acute kidney injury by inhibition of uremic toxins such as indoxyl sulfate and pcresol sulfate via alleviating dysbiosis. Int. J. Mol. Med..

[27] Lee Y.J., Li K.Y., Wang P.J., Huang H.W., Chen M.J. (2020). Alleviating chronic kidney disease progression through modulating the critical genus of gut microbiota in a cisplatin-induced Lanyu pig model. J. Food Drug Anal..

[28] Li J., Sung C.Y., Lee N., Ni Y., Pihlajamaki J., Panagiotou G., El-Nezami H. (2016). Probiotics modulated gut microbiota suppresses hepatocellular carcinoma growth in mice. Proc. Natl. Acad. Sci. USA.

[29] Li S., Qi C., Zhu H., Yu R., Xie C., Peng Y., Yin S.W., Fan J., Zhao S., Sun J. (2019). Lactobacillus reuteri improves gut barrier function and affects diurnal variation of the gut microbiota in mice fed a high-fat diet. Food Funct..

[30] Li X.W., Feng L.X., Zhu X.J., Liu Q., Wang H.S., Wu X., Yan P., Duan X.J., Xiao Y.Q., Cheng W. (2020). Human umbilical cord blood mononuclear cells protect against renal tubulointerstitial fibrosis in cisplatin-treated rats. Biomed. Pharmacother..

[31] Liu H.X., Rocha C.S., Dandekar S., Wan Y.J. (2016). Functional analysis of the relationship between intestinal microbiota and the expression of hepatic genes and pathways during the course of liver regeneration. J. Hepatol..

[32] Liu S.P., Chang C.Y., Huang W.H., Fu Y.S., Chao D., Huang H.T. (2010). Dimethylthiourea pretreatment inhibits endotoxin-induced compound exocytosis in goblet cells and plasma leakage of rat small intestine. J. Electron. Microsc..

[33] Machado R.A., Constantino Lde S., Tomasi C.D., Rojas H.A., Vuolo F.S., Vitto M.F., Cesconetto P.A., de Souza C.T., Ritter C., Dal-Pizzol F. (2012). Sodium butyrate decreases the activation of NF-kappaB reducing inflammation and oxidative damage in the kidney of rats subjected to contrast-induced nephropathy. Nephrol. Dial. Transplant.

[34] Machiels K., Joossens M., Sabino J., De Preter V., Arijs I., Eeckhaut V., Ballet V., Claes K., Van Immerseel F., Verbeke K. (2014). A decrease of the butyrate-producing species Roseburia hominis and Faecalibacterium prausnitzii defines dysbiosis in patients with ulcerative colitis. Gut.

[35] Marietta E., Horwath I., Taneja V. (2018). Microbiome, Immunomodulation, and the Neuronal System. Neurotherapeutics.

[36] Montassier E., Gastinne T., Vangay P., Al-Ghalith G.A., Bruley des Varannes S., Massart S., Moreau P., Potel G., de La Cochetiere M.F., Batard E. (2015). Chemotherapy-driven dysbiosis in the intestinal microbiome. Aliment. Pharmacol. Ther..

[37] Mu Q., Tavella V.J., Luo X.M. (2018). Role of Lactobacillus reuteri in Human Health and Diseases. Front. Microbiol..

[38] Oka K., Osaki T., Hanawa T., Kurata S., Sugiyama E., Takahashi M., Tanaka M., Taguchi H., Kamiya S. (2018). Establishment of an Endogenous Clostridium difficile Rat Infection Model and Evaluation of the Effects of Clostridium butyricum MIYAIRI 588 Probiotic Strain. Front. Microbiol..

[39] Pan H., Li J., Rankin G.O., Rojanasakul Y., Tu Y., Chen Y.C. (2018). Synergistic effect of black tea polyphenol, theaflavin-3,3’-digallate with cisplatin against cisplatin resistant human ovarian cancer cells. J. Funct. Foods.

[40] Perales-Puchalt A., Perez-Sanz J., Payne K.K., Svoronos N., Allegrezza M.J., Chaurio R.A., Anadon C., Calmette J., Biswas S., Mine J.A. (2018). Frontline Science: Microbiota reconstitution restores intestinal integrity after cisplatin therapy. J. Leukoc. Biol..

[41] Pianta T.J., Pickering J.W., Succar L., Chin M., Davidson T., Buckley N.A., Mohamed F., Endre Z.H. (2017). Dexamethasone Modifies Cystatin C-Based Diagnosis of Acute Kidney Injury During Cisplatin-Based Chemotherapy. Kidney Blood Press. Res..

[42] Plaza-Diaz J., Ruiz-Ojeda F.J., Vilchez-Padial L.M., Gil A. (2017). Evidence of the Anti-Inflammatory Effects of Probiotics and Synbiotics in Intestinal Chronic Diseases. Nutrients.

[43] Li L., Fang Z., Liu X., Hu W., Lu W., Lee Y.K., Zhao J., Zhang H., Chen W. (2020). Lactobacillus reuteri attenuated allergic inflammation induced by HDM in the mouse and modulated gut microbes. PLoS ONE.

[44] Ren X., Liu L., Liu P., Gamallat Y., Xin Y., Shang D. (2018). Polysaccharide extracted from Enteromorpha ameliorates Cisplastininduced small intestine injury in mice. J. Funct. Foods.

[45] Rescigno M. (2014). Intestinal microbiota and its effects on the immune system. Cell Microbiol..

[46] Schnackenberg L.K., Sun J., Pence L.M., Bhattacharyya S., Gamboa da Costa G., Beger R.D. (2012). Metabolomics evaluation of hydroxyproline as a potential marker of melamine and cyanuric acid nephrotoxicity in male and female Fischer F344 rats. Food Chem. Toxicol..

[47] Schroeder B.O., Birchenough G.M.H., Stahlman M., Arike L., Johansson M.E.V., Hansson G.C., Backhed F. (2018). Bifidobacteria or Fiber Protects against Diet-Induced Microbiota-Mediated Colonic Mucus Deterioration. Cell Host Microbe.

[48] Seki H., Shiohara M., Matsumura T., Miyagawa N., Tanaka M., Komiyama A., Kurata S. (2003). Prevention of antibiotic-associated diarrhea in children by Clostridium butyricum MIYAIRI. Pediatr. Int..

[49] Seo M., Inoue I., Tanaka M., Matsuda N., Nakano T., Awata T., Katayama S., Alpers D.H., Komoda T. (2013). Clostridium butyricum MIYAIRI 588 improves high-fat diet-induced non-alcoholic fatty liver disease in rats. Dig. Dis. Sci..

[50] Shackelford C., Long G., Wolf J., Okerberg C., Herbert R. (2002). Qualitative and quantitative analysis of nonneoplastic lesions in toxicology studies. Toxicol. Pathol..

[51] Sharp C.N., Doll M.A., Dupre T.V., Shah P.P., Subathra M., Siow D., Arteel G.E., Megyesi J., Beverly L.J., Siskind L.J. (2016). Repeated administration of low-dose cisplatin in mice induces fibrosis. Am. J. Physiol. Renal. Physiol..

[52] Shen F., Zheng R.D., Sun X.Q., Ding W.J., Wang X.Y., Fan J.G. (2017). Gut microbiota dysbiosis in patients with non-alcoholic fatty liver disease. Hepatobiliary Pancreat. Dis. Int..

[53] Shinnoh M., Horinaka M., Yasuda T., Yoshikawa S., Morita M., Yamada T., Miki T., Sakai T. (2013). Clostridium butyricum MIYAIRI 588 shows antitumor effects by enhancing the release of TRAIL from neutrophils through MMP-8. Int. J. Oncol..

[54] Takayama F., Taki K., Niwa T. (2003). Bifidobacterium in gastro-resistant seamless capsule reduces serum levels of indoxyl sulfate in patients on hemodialysis. Am. J. Kidney Dis..

[55] Tang Y., Wu Y., Huang Z., Dong W., Deng Y., Wang F., Li M., Yuan J. (2017). Administration of probiotic mixture DM#1 ameliorated 5-fluorouracil-induced intestinal mucositis and dysbiosis in rats. Nutrition.

[56] Tian T., Xu B., Qin Y., Fan L., Chen J., Zheng P., Gong X., Wang H., Bai M., Pu J. (2019). Clostridium butyricum miyairi 588 has preventive effects on chronic social defeat stress-induced depressive-like behaviour and modulates microglial activation in mice. Biochem. Biophys. Res. Commun..

[57] Wen J.J., Vyatkina G., Garg N. (2004). Oxidative damage during chagasic cardiomyopathy development: Role of mitochondrial oxidant release and inefficient antioxidant defense. Free Radic. Biol. Med..

[58] Wong J., Piceno Y.M., DeSantis T.Z., Pahl M., Andersen G.L., Vaziri N.D. (2014). Expansion of urease- and uricase-containing, indole- and p-cresol-forming and contraction of short-chain fatty acid-producing intestinal microbiota in ESRD. Am. J. Nephrol..

[59] Wong Y.S., Lin M.Y., Liu P.F., Ko J.L., Huang G.T., Tu D.G., Ou C.C. (2020). D-methionine improves cisplatin-induced anorexia and dyspepsia syndrome by attenuating intestinal tryptophan hydroxylase 1 activity and increasing plasma leptin concentration. Neurogastroenterol. Motil..

[60] Wu C.H., Ko J.L., Liao J.M., Huang S.S., Lin M.Y., Lee L.H., Chang L.Y., Ou C.C. (2019). D-methionine alleviates cisplatin-induced mucositis by restoring the gut microbiota structure and improving intestinal inflammation. Ther. Adv. Med. Oncol..

[61] Wu I.W., Lin C.Y., Chang L.C., Lee C.C., Chiu C.Y., Hsu H.J., Sun C.Y., Chen Y.C., Kuo Y.L., Yang C.W. (2020). Gut Microbiota as Diagnostic Tools for Mirroring Disease Progression and Circulating Nephrotoxin Levels in Chronic Kidney Disease: Discovery and Validation Study. Int. J. Biol. Sci..

[62] Xu K.Y., Xia G.H., Lu J.Q., Chen M.X., Zhen X., Wang S., You C., Nie J., Zhou H.W., Yin J. (2017). Impaired renal function and dysbiosis of gut microbiota contribute to increased trimethylamine-N-oxide in chronic kidney disease patients. Sci. Rep..

[63] Yang B., El Nahas A.M., Thomas G.L., Haylor J.L., Watson P.F., Wagner B., Johnson T.S. (2001). Caspase-3 and apoptosis in experimental chronic renal scarring. Kidney Int..

[64] Ye J., Lv L., Wu W., Li Y., Shi D., Fang D., Guo F., Jiang H., Yan R., Ye W. (2018). Butyrate Protects Mice Against Methionine-Choline-Deficient Diet-Induced Non-alcoholic Steatohepatitis by Improving Gut Barrier Function, Attenuating Inflammation and Reducing Endotoxin Levels. Front. Microbiol..

[65] Yen C.H., Tseng Y.H., Kuo Y.W., Lee M.C., Chen H.L. (2011). Long-term supplementation of isomalto-oligosaccharides improved colonic microflora profile, bowel function, and blood cholesterol levels in constipated elderly people--a placebo-controlled, diet-controlled trial. Nutrition.

[66] Yoshifuji A., Wakino S., Irie J., Tajima T., Hasegawa K., Kanda T., Tokuyama H., Hayashi K., Itoh H. (2016). Gut Lactobacillus protects against the progression of renal damage by modulating the gut environment in rats. Nephrol. Dial. Transplant.

[67] Zhai S., Qin S., Li L., Zhu L., Zou Z., Wang L. (2019). Dietary butyrate suppresses inflammation through modulating gut microbiota in high-fat diet-fed mice. FEMS Microbiol. Lett..

[68] Zhao L., Zhang Q., Ma W., Tian F., Shen H., Zhou M. (2017). A combination of quercetin and resveratrol reduces obesity in high-fat diet-fed rats by modulation of gut microbiota. Food Funct..

[69] Zhou P., Li Z., Xu D., Wang Y., Bai Q., Feng Y., Su G., Chen P., Wang Y., Liu H. (2019). Cepharanthine Hydrochloride Improves Cisplatin Chemotherapy and Enhances Immunity by Regulating Intestinal Microbes in Mice. Front. Cell Infect. Microbiol..

