# Supplementary material for: Administration of Lactobacillus reuteri Combined with Clostridium butyricum Attenuates Cisplatin-Induced Renal Damage by Gut Microbiota Reconstitution, Increasing Butyric Acid Production, and Suppressing Renal Inflammation
Source: Nutrients. 2021 Aug 15;13(8):2792. doi: 10.3390/nu13082792 (PMC8402234; doi:10.3390/nu13082792)
Supplement: Supplementary file 1 [file nutrients-13-02792-s001.zip › nutrients-1313118-supplementary.pdf]

Administration of *Lactobacillus reuteri* combined with *Clostridium butyricum* attenuates cisplatin-induced renal damage by gut microbiota reconstitution, increasing butyric acid production, and suppressing renal inflammation

Supplementary Tables and Figures

Table S1 Effect of cisplatin and probiotics on organ weights and relative tissue weights

(A) Organ weights

	Control	Cisplatin	Cis+LCs	LCs
Liver (g)	13.5±0.44	9.46±0.05*	8.61±0.39*	12.51±0.18#
Spleen (g)	0.96±0.09	0.62±0.07*	0.60±0.06*	0.92±0.06#
Kidney (g)	3.08±0.22	4.91±0.54*	2.96±0.29#	2.67±0.10#
Stomach (g)	1.91±0.03	1.62±0.08*	1.72±0.06	1.83±0.05#

(B) Relative tissue weight

	Control	Cisplatin	Cis+LCs	LCs
Liver/BW ratio (%)	3.51±0.07	3.72±0.05	3.44±0.03#	3.35±0.08#
Spleen/BW ratio (%)	0.25±0.02	0.24±0.02	0.24±0.02	0.25±0.02
Kidney/BW ratio (%)	0.80±0.06	2.16±0.30*	1.15±0.15#	0.71±0.02#
Stomach/BW ratio (%)	0.50±0.01	0.63±0.01*	0.69±0.01*#	0.49±0.01#

Results are shown as mean ± SEM (n = 7 per group). *$p < 0.05$ compared with the control group; #$p < 0.05$ compared with the cisplatin group.

Table S2 The pH, moisture content, wet and dry weights of stool after the treatment of cisplatin and probiotics

Stool	Control	Cisplatin	Cis+LCs	LCs
Wet weight (g)	12.24±1.5	3.83±0.72*	9.29±1.68#	12.40±1.15#
Dry Weight (g)	4.66±0.50	1.75±0.29*	3.52±0.51#	4.41±0.29#
Moisture content (%)	60.42±2.69	53.09±1.54	59.45±2.63	63.60±1.88#
pH	7.20±0.16	7.20±.10	7.27±0.08	7.33±0.28

Results are shown as mean ± SEM (n = 7 per group). *$p < 0.05$ compared with the control group; #$p < 0.05$ compared with the cisplatin group.

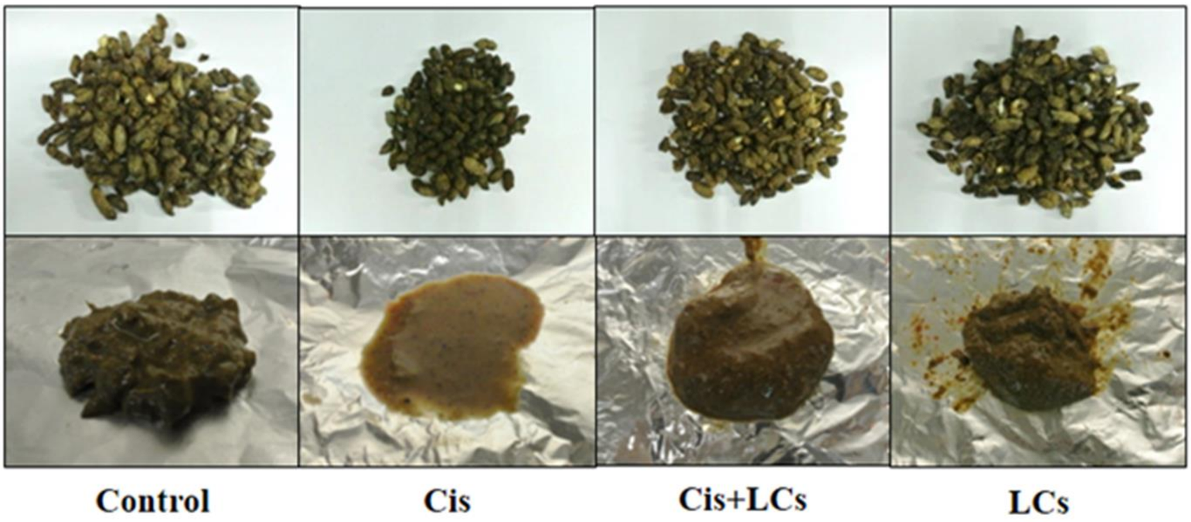

(A)

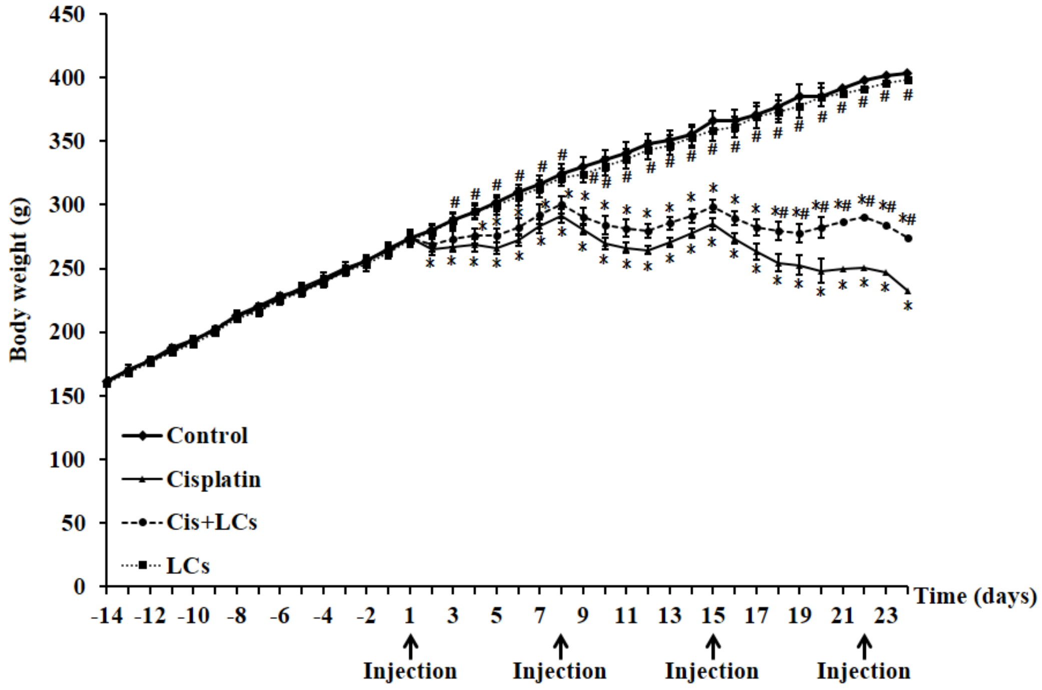
Body weight (g)
Time (days)
Control
Cisplatin
Cis+LCs
LCs
Injection
Injection
Injection
Injection

(B)

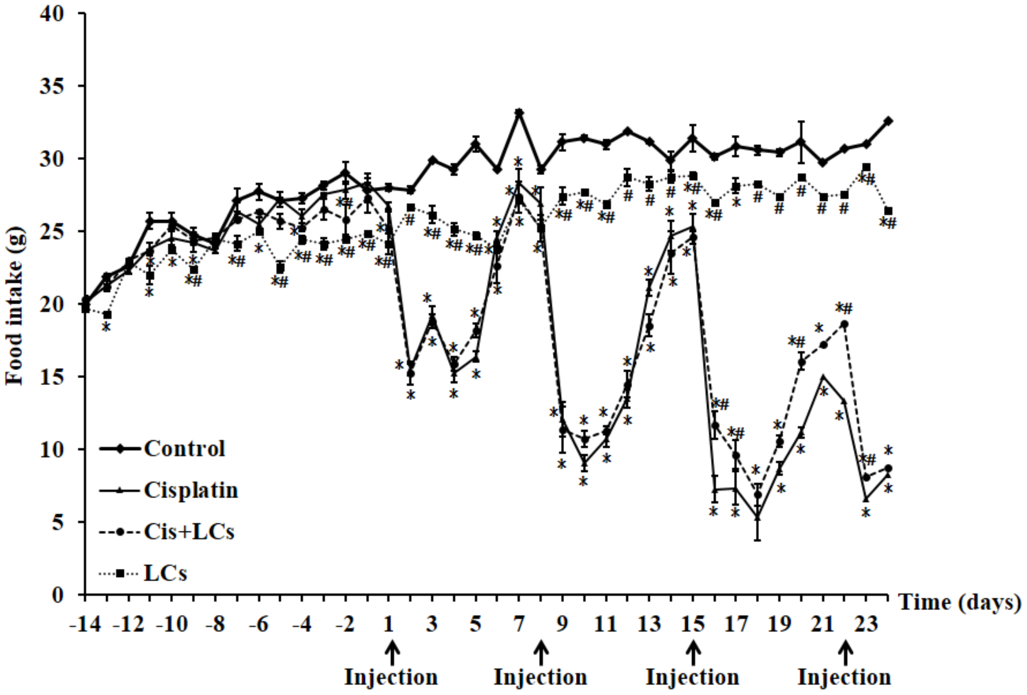
Food intake (g)
Time (days)
Control
Cisplatin
Cis+LCs
LCs
Injection
Injection
Injection
Injection

(C)

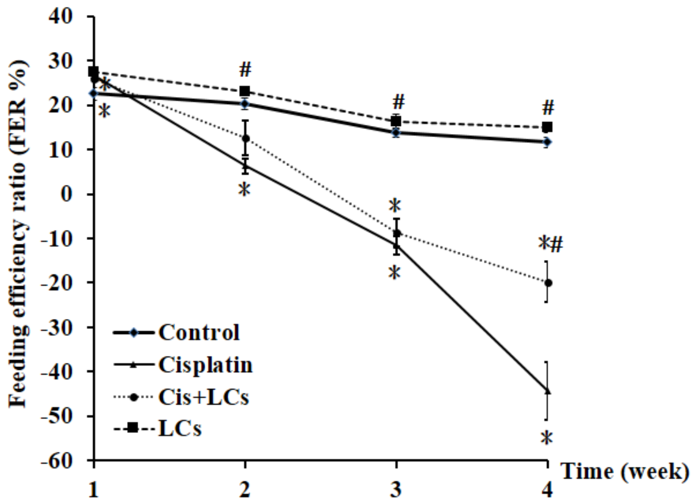
Feeding efficiency ratio (FER %)
40
30
20
10
0
-10
-20
-30
-40
-50
-60
1
2
3
4
Time (week)
Control
Cisplatin
Cis+LCs
LCs
#
*
*#

(D)

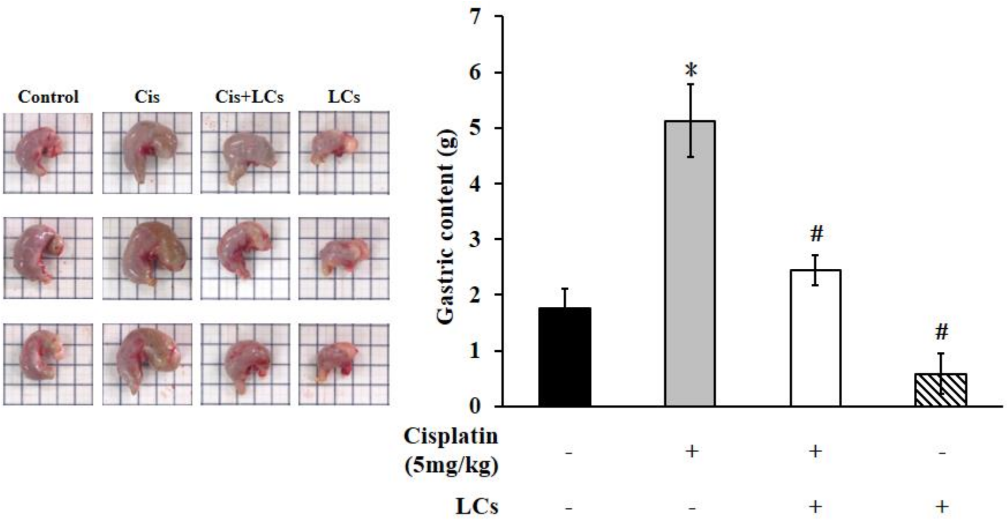
Control
Cis
Cis+LCs
LCs
Gastric content (g)
7
6
5
4
3
2
1
0
*
#
Cisplatin
(5mg/kg)
- + + -
LCs
- - + +

(E)

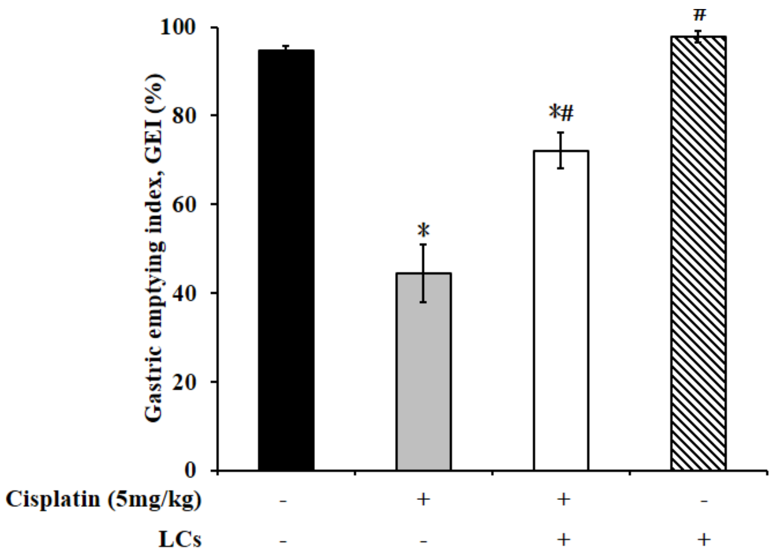

Figure S1. Effects of cisplatin and LCs supplementation on digestive function. (A) Body weight changes during the experimental period. (B) Food intake changes during the experimental period. (C) Feeding efficiency ratio. (D) Gastric content. (E) Gastric emptying index. Results are shown as mean ± SEM (n = 7 per group). *$p < 0.05$ compared with the control group; #$p < 0.05$ compared with the cisplatin group.

(A)

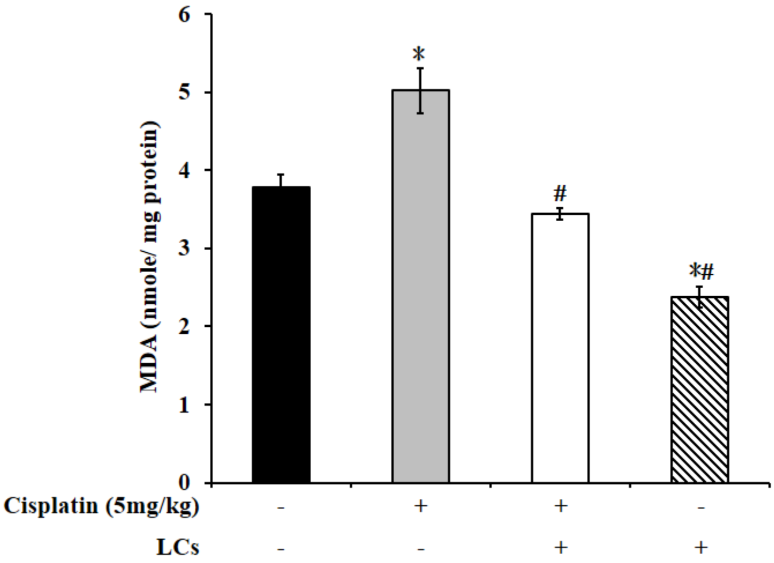

MDA (nmole/ mg protein)
0
1
2
3
4
5
6
*
#
*#
Cisplatin (5mg/kg) - + + -
LCs - - + +

(B)

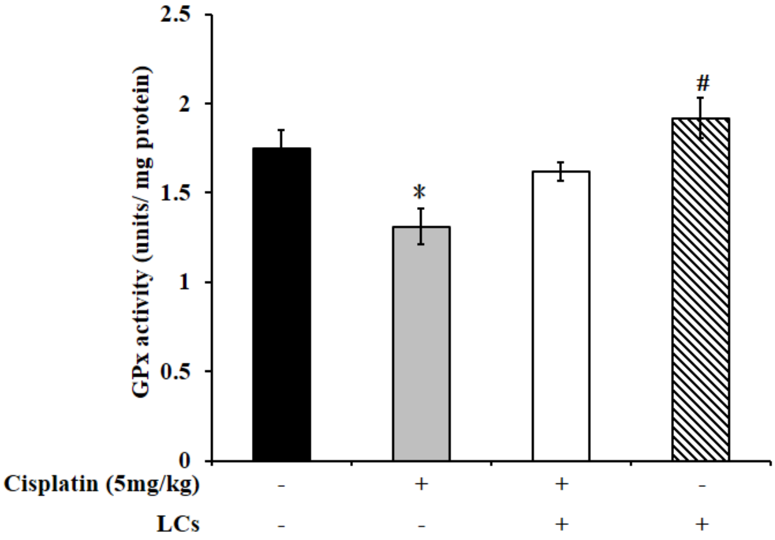

GPx activity (units/ mg protein)
0
0.5
1
1.5
2
2.5
*
#
Cisplatin (5mg/kg) - + + -
LCs - - + +

(C)

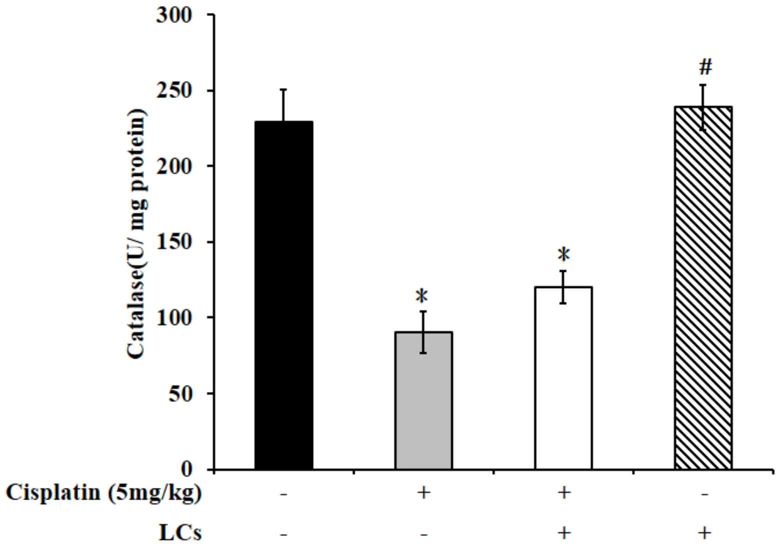

Figure S2. Effects of cisplatin and LCs supplementation on antioxidant/oxidant status in kidney tissues. (A) MDA. (B) GPx activity. (C) Catalase. Results are shown as mean ± SEM (n = 7 per group). *$p < 0.05$ compared with the control group; #$p < 0.05$ compared with the cisplatin group.
